# COVID-19 Vaccine Uptake in Immigrant, Refugee, and Nonimmigrant Children and Adolescents in Ontario, Canada

**DOI:** 10.1001/jamanetworkopen.2023.25636

**Published:** 2023-07-26

**Authors:** Julia Brandenberger, Raquel Duchen, Hong Lu, Susitha Wanigaratne, Eyal Cohen, Teresa To, Pierre-Philippe Piché-Renaud, Astrid Guttmann

**Affiliations:** 1 Edwin S.H. Leong Centre for Healthy Children, University of Toronto, Toronto, Ontario, Canada; 2 Division of Pediatric Emergency Medicine, Hospital for Sick Children, Toronto, Ontario, Canada; 3 Pediatric Emergency Department, University Hospital of Bern, Bern, Switzerland; 4 Child Health Evaluative Sciences, SickKids Research Institute, Toronto, Ontario, Canada; 5 ICES, Toronto, Ontario, Canada; 6 Division of Paediatric Medicine, Hospital for Sick Children, Toronto, Ontario, Canada; 7 Department of Paediatrics, University of Toronto, Toronto, Ontario, Canada; 8 Division of Pediatric Infectious Diseases, The Hospital for Sick Children, Toronto, Ontario, Canada; 9 Dalla Lana School of Public Health, University of Toronto, Toronto, Ontario, Canada

## Abstract

**Importance:**

COVID-19 vaccinations are recommended for minors. Surveys indicate lower vaccine acceptance by some immigrant and refugee groups.

**Objective:**

To identify characteristics in immigrant, refugee, and nonimmigrant minors associated with vaccination.

**Design, Setting, and Participants:**

This retrospective cohort study used linked, population-based demographic and health care data from Ontario, Canada, including all children aged 4 to 17 years registered for universal health insurance on January 1, 2021, across 2 distinct campaigns: for adolescents (ages 12-17 years), starting May 23, 2021, and for children (ages 5-11 years), starting November 25, 2021, through April 24, 2022. Data were analyzed from May 9 to August 2, 2022.

**Exposures:**

Immigrant or refugee status and immigration characteristics (recency, category, region of origin, and generation).

**Main Outcomes and Measures:**

Outcomes of interest were crude rates of COVID-19 vaccination (defined as ≥1 vaccination for children and ≥2 vaccinations for adolescents) and adjusted odds ratios (aORs) with 95% CIs for vaccination, adjusted for clinical, sociodemographic, and health system factors.

**Results:**

The total cohort included 2.2 million children and adolescents, with 1 098 749 children (mean [SD] age, 7.06 [2.00] years; 563 388 [51.3%] males) and 1 142 429 adolescents (mean [SD] age, 14.00 [1.99] years; 586 617 [51.3%] males). Among children, 53 090 (4.8%) were first-generation and 256 886 (23.4%) were second-generation immigrants or refugees; among adolescents, 104 975 (9.2%) were first-generation and 221 981 (19.4%) were second-generation immigrants or refugees, most being economic or family-class immigrants. Immigrants, particularly refugees, were more likely to live in neighborhoods with highest material deprivation (first-generation immigrants: 18.6% of children and 20.2% of adolescents; first-generation refugees: 46.4% of children and 46.3% of adolescents; nonimmigrants: 18.5% of children and 17.2% of adolescents) and COVID-19 risk (first-generation immigrants; 20.0% of children and 20.5% of adolescents; first-generation refugees: 9.4% of children and 12.6% of adolescents; nonimmigrants: 6.9% of children and 6.8% of adolescents). Vaccination rates (53.1% in children and 79.2% in adolescents) were negatively associated with material deprivation. In both age groups, odds for vaccination were higher in immigrants (children: aOR, 1.30; 95% CI, 1.27-1.33; adolescents: aOR, 1.10; 95% CI, 1.08-1.12) but lower in refugees (children: aOR, 0.34; 95% CI, 0.33-0.36; adolescents: aOR, 0.88; 95% CI, 0.84-0.91) compared with nonimmigrants. In immigrant- and refugee-only models stratified by generation, region of origin was associated with uptake, compared with the overall rate, with the lowest odds observed in immigrants and refugees from Eastern Europe (children: aOR, 0.40; 95% CI, 0.35-0.46; adolescents: aOR, 0.41; 95% CI, 0.38-0.43) and Central Africa (children: aOR, 0.24; 95% CI, 0.16-0.35; adolescents: aOR, 0.51,CI: 0.45-0.59) and the highest odds observed in immigrants and refugees from Southeast Asia (children: aOR, 2.68; 95% CI, 2.47-2.92; adolescents aOR, 4.42; 95% CI, 4.10-4.77). Adjusted odds of vaccination among immigrants and refugees from regions with lowest vaccine coverage were similar across generations.

**Conclusions and Relevance:**

In this cohort study using a population-based sample in Canada, nonrefugee immigrants had higher vaccine coverage than nonimmigrants. Substantial heterogeneity by region of origin and lower vaccination coverage in refugees persisted across generations. These findings suggest that vaccine campaigns need precision public health approaches targeting specific barriers in identified, undervaccinated subgroups.

## Key Points

**Question:**

What are the COVID-19 vaccination rates in immigrant and refugee children (5-11 years) and adolescents (12-17 years) in Ontario, Canada?

**Findings:**

In this cohort study of 2.2 million minors, vaccine coverage was 53.1% for children (≥1 dose) and 79.2% for adolescents (≥2 doses), and uptake was higher in immigrants and lower in refugees compared with nonimmigrants. There was significant heterogeneity by region of origin in first- and second-generation immigrants and refugees, even after adjusting for immigration category and other sociodemographic factors.

**Meaning:**

These findings suggest that precision public health approaches are warranted to increase vaccination in some immigrant, and particularly refugee, subgroups.

## Teaser

This cohort study examines characteristics associated with COVID-19 vaccination in immigrant, refugee, and nonimmigrant children and adolescents in Ontario, Canada.

## Introduction

COVID-19 is the eighth leading cause of death in minors in the US^1^ and has negatively affected pediatric populations worldwide.^2,3,4,5^ Vaccination of children can mitigate risk of complications from SARS-CoV-2 infections^6^ and support their uninterrupted access to education and healthy psychosocial development.^7,8,9^ US and Canadian public health guidance strongly recommends COVID-19 vaccinations for minors.^10,11^

Despite adequate supply and accessibility, studies report parents are less likely to vaccinate their children than themselves.^12,13,14^ Factors associated with parents’ willingness to vaccinate children include older age of parents or guardians, good access to scientific information, and acceptance of routine and influenza vaccinations.^15^ A systematic review and meta-analysis of 44 cross-sectional surveys described that while 60.1% of parents intended to vaccinate their children against COVID-19, the range was very wide (25.6%-92.2%).^15^ Diverse cultural and religious beliefs, education, and experiences with the health system may be important factors, especially in countries with large immigrant populations. A systematic review on vaccination determinants among migrants in Europe showed undervaccination among adults was associated with geographic origin.^16^ It is unclear whether these trends persist in migrants living in other countries, with different immigration selection criteria, social supports, and public health practices. North American surveys describing parental intention to vaccinate minors against COVID-19 have highlighted higher vaccine hesitancy in racialized populations, such as individuals identifying as Arab, Black, Hispanic, or Latin American.^17,18,19^ To our knowledge, there have been few empirical or population-based studies on COVID-19 vaccine coverage in minors^20^ and none have explored vaccination in immigrant and refugee populations in North America.

Health Canada authorized a COVID-19 vaccine for persons aged at least 16 years in December 2020, for adolescents (ages 12-15 years) in May 2021^21^ and for children (ages 5-11 years) in November 2021^22,23,24^ (eFigure 1 in Supplement 1). Adolescents were part of an initial campaign; however high-risk populations, such as health care workers and older individuals, were initially prioritized. The campaign had a strong focus on vaccine equity. With constraints in vaccine availability during the first month of the adolescent vaccination campaign, 50% of available vaccines were allocated to 30% of neighborhoods that had high rates of COVID-19–related hospitalizations^25^ and were made up of highly racially and ethnically diverse populations, many of whom were immigrants and essential workers.^26^ The $1.5 billion campaign, mainly funded by the provincial government,^27^ leveraged numerous community-led approaches that were culturally and linguistically tailored to reduce barriers,^28,29^ including mobile clinics and door-to-door campaigns.^30,31^ The children’s vaccination campaign had less focus on high-risk communities^32^; however, vaccine supply was adequate by that time.

Ontario is Canada’s largest province (40% of the total population), with a racially and ethnically diverse population, with 2.9% identifying as Indigenous,^33^ and 30% identifying as immigrants, of whom approximately 70% are racialized.^34^ Canada’s immigration system primarily selects economic immigrants with high levels of education, language ability, and work experience. However, immigration pathways exist for family members, resettled refugees, and asylum seekers. Like nonimmigrants, all successful asylum seekers and refugees receive provincial health insurance coverage. In Ontario, nonrefugee immigrants become eligible 3 months after arrival, and refugees are immediately insured either through the provincial insurance (resettled refugees) or a temporary federal program (asylum seekers). Undocumented migrants are not covered but have access to some free primary care through community health centers.^35^ All Ontario residents are entitled to free COVID-19 vaccinations.

This study capitalized on linked, population-based health care and demographic data to analyze vaccination in minors in Ontario across the 2 distinct campaigns, focusing on immigrants and refugees. Given the stronger equity focus in the adolescent campaign,^25^ we hypothesized that COVID-19 vaccination coverage would be higher and more homogenous across socioeconomic and immigrant characteristics in adolescents than in younger children. We also postulated that there would be significant differences between immigrant and refugee groups once disaggregated by immigration category and region of origin, but that these associations would be moderated in the second generation, associated with acculturation, as described by others.^16^

## Methods

### Study Design

This retrospective population-based cohort study used encoded data from multiple, linked data sets, including the provincial health insurance registry, the COVID-19 vaccine registry, and the federal immigration permanent resident files (eTable 1 in Supplement 1), accessed at ICES.^36^ ICES is an independent, nonprofit research institute whose legal status under Ontario’s health information privacy law allows collection and analysis of health care data, without consent, for health system evaluation. The use of data in this project was authorized under section 45 of Ontario’s Personal Health Information Protection Act, thus not requiring research ethics board approval. The study followed Strengthening the Reporting of Observational Studies in Epidemiology (STROBE) and Reporting of Studies Conducted Using Observational Routinely-Collected Health Data (RECORD) reporting guidelines.

This study included all children aged 4 to 17 years living in Ontario, Canada, on January 1, 2021, and registered in the universal Ontario Health Insurance Program^37^ for at least 1 year. The cohort was stratified to reflect the 2 distinct vaccine campaigns in 2021, beginning May 23 for adolescents^23^ and November 25 for children.^22^ Each participant was assigned an individual eligibility date based on their birthday (ie, children aged 4 years became eligible on their fifth birthday) and followed until the end of the observation window (April 24, 2022) (eFigure 1 in Supplement 1).

### Outcome Measures and Exposures

Vaccination was defined as receipt of at least 1 dose of a COVID-19 vaccine for children (ages 5-11 years) and at least 2 doses for adolescents (ages 12-17 years) (eTable 1 and eTable 2 in Supplement 1). We used 1 dose for children as there were only 5 months between the start of their campaign and study end. Additionally, there were recommendations to delay doses at least 3 months after SARS-CoV-2 infection.^38^ While SARS-CoV-2 polymerase chain reaction testing in Ontario was limited due to overwhelming case volumes after November 2021,^39,40^ surveillance studies from other Canadian jurisdictions suggest high pediatric infection rates during that time.^41^

Main exposures of interest were immigration characteristics: category (economic or family-sponsored immigrant, resettled refugee [fulfilling the United Nations High Commissioner for Refugees definition prior to arrival in Canada], or protected person [refugee who successfully applied for asylum through the in-country immigration pathway) (eTable 3 in Supplement 1), time since immigration, region of origin, and generation. First-generation immigrants and refugees were identified by the immigration database. Linked maternal- or birthing parent–infant hospital delivery records were used to assign the immigration characteristics of mothers and birthing parents to their second-generation children (eTable 1 and eTable 2 in Supplement 1). All others were categorized as nonimmigrant minors.

### Covariates

Baseline characteristics were recorded on January 1, 2021 (eTable 1 in Supplement 1). Individual-level covariates hypothesized to be associated with vaccination included age, sex, having a pediatric chronic condition, influenza vaccination in 2019 or 2020, primary care model (family practice, pediatrician, community health center, or no regular primary care practitioner), and history of SARS-CoV-2 infection. Socioeconomic characteristics have been reported to be associated with vaccination and are potential mediators between immigrant or refugee status and vaccination.^42^ We used the material deprivation construct from the census-based Ontario Marginalization Index,^43^ which includes income and education information on a neighborhood level to capture socioeconomic disparities, and previously derived deciles of neighborhood COVID-19 risk^44^ based on COVID-19 cases from the beginning of the pandemic until March 23, 2021 (eTable 2 in Supplement 1).^25^

### Statistical Analysis

We compared baseline characteristics of both generations of immigrants and refugees with nonimmigrants using standardized differences (>0.1 signified important differences).^45^ Given the size of the cohort, we did not test differences in crude rates but commented on clinically important differences.

Using logistic regression, we first modeled the association of immigrant category (with nonimmigrants as the reference group) with vaccination in the full study population and included all covariates. To understand the associations among immigration characteristics, we did a subgroup analysis of first- and second-generation immigrants and refugees, stratified by generation, as we hypothesized different associations by generation. We included key mediators, like socioeconomic characteristics (material deprivation quintile) but did not include influenza vaccination and previous SARS-CoV-2 infection, as these models were intended to test which intersecting sociodemographic and immigration characteristics were most strongly associated with vaccination. We compared differences within each generation using 95% CIs of adjusted odds ratios (aORs). To explore if associations between the exposures and vaccine hesitancy were different than those for vaccine uptake, we performed a secondary analysis using time-to-event models to calculate hazard ratios (HRs) associated with first doses. Individuals with missing or suppressed data were merged to the most appropriate category or excluded from the final models (eTable 2 in Supplement 1). Statistical analyses were conducted using SAS statistical software, Enterprise Guide version 7.1 (SAS Institute). Data were analyzed from May 9 to August 2, 2022.

## Results

Approximately 2.2 million Ontario minors were included in the study, with nearly equal numbers of children (1 098 749 children; mean [SD] age, 7.06 [2.00] years; 563 388 [51.3%] males) and adolescents (1 142 429 adolescents; mean [SD] age, 14.00 [1.99] years; 586 617 [51.3%] males) (eFigure 2 in Supplement 1). Of the children’s cohort, 53 090 children (4.8%) were first-generation immigrants or refugees and 256 886 children (23.4%) were second-generation immigrants or refugees. In adolescents, 104 975 adolescents (9.2%) were first-generation immigrants or refugees and 221 981 adolescents (19.4%) were second-generation immigrants or refugees. In both cohorts, regions of origin of immigrants and refugees were mostly South Asia (15 931 children [30.0%]; 25 209 adolescents [24.0%]) and the Middle East (12 732 children [24.0%]; 22 503 adolescents [21.4%]). Second-generation minors most frequently had mothers or birthing parents originating from South Asia (Table 1). Immigrants, and particularly refugees, were more likely than nonimmigrants to live in neighborhoods with highest material deprivation (first-generation immigrants: 18.6% of children and 20.2% of adolescents; first-generation refugees: 46.4% of children and 46.3% of adolescents; nonimmigrants: 18.5% of children and 17.2% of adolescents) (Table 1; eTable 4 in Supplement 1).

**Table 1.** Baseline Characteristics of Immigrants, Refugees, and Nonimmigrant Children and Adolescents in Ontario on January 1, 2021^a^

Characteristic	First-generation immigrants and refugees					Second-generation immigrants and refugees^b^						Nonimmigrants	
	No. (column %)			Standardized difference^c^		No. (column %)			Standardized difference^c^			No. (column %)	Standardized difference^c^
	Immigrants^d^	Refugees^e^	Protected persons^f^	Refugees	Protected persons	Immigrants	Refugees	Protected persons	Immigrants	Refugees	Protected persons		
**Children**													
Total, No.	36 584	10 144	6362	NA	NA	218 093	14 800	23 993	NA	NA	NA	788 773	NA
Age, y													
Mean (SD)	7.55 (1.92)	7.51 (1.86)	7.83 (1.79)	0.017	0.153	7.01 (2.00)	6.85 (2.01)	6.90 (2.00)	0.275	0.352	0.328	7.04 (2.00)	0.258
4-7	16 524 (45.2)	4762 (46.9)	2532 (39.8)	0.036	0.109	123 868 (56.8)	8859 (59.9)	14 181 (59.1)	0.234	0.297	0.282	443 300 (56.2)	0.222
8-10	20 060 (54.8)	5382 (53.1)	3830 (60.2)	0.036	0.109	94 225 (43.2)	5941 (40.1)	9812 (40.9)	0.234	0.297	0.282	345 473 (43.8)	0.222
Sex													
Female	17 868 (48.8)	4973 (49.0)	3202 (50.3)	0.004	0.030	106 089 (48.6)	7280 (49.2)	11 780 (49.1)	0.004	0.007	0.005	384 169 (48.7)	0.003
Male	18 716 (51.2)	5171 (51.0)	3160 (49.7)	0.004	0.030	112 004 (51.4)	7520 (50.8)	12 213 (50.9)	0.004	0.007	0.005	404 604 (51.3)	0.003
Material deprivation quintile													
1 (Least deprived)	8649 (23.6)	730 (7.2)	697 (11.0)	0.468	0.34	49 956 (22.9)	2940 (19.9)	3668 (15.3)	0.017	0.092	0.212	201 221 (25.5)	0.043
2	7604 (20.8)	1023 (10.1)	865 (13.6)	0.299	0.191	46 471 (21.3)	2643 (17.9)	3895 (16.2)	0.013	0.074	0.117	173 353 (22.0)	0.029
3	6858 (18.7)	1528 (15.1)	878 (13.8)	0.098	0.134	41 056 (18.8)	2261 (15.3)	4001 (16.7)	0.002	0.092	0.054	142 164 (18.0)	0.019
4	6674 (18.2)	2160 (21.3)	1311 (20.6)	0.077	0.06	38 719 (17.8)	2286 (15.4)	4604 (19.2)	0.013	0.075	0.024	125 941 (16.0)	0.06
5 (Most deprived)	6799 (18.6)	4703 (46.4)	2611 (41.0)	0.621	0.506	41 891 (19.2)	4670 (31.6)	7825 (32.6)	0.016	0.303	0.326	146 094 (18.5)	0.002
Region of origin													
Central Africa	174 (0.5)	88 (0.9)	122 (1.9)	0.048	0.133	715 (0.3)	304 (2.1)	743 (3.1)	0.023	0.142	0.199	NA	NA
Western Africa	1194 (3.3)	49 (0.5)	423 (6.6)	0.206	0.156	4678 (2.1)	246 (1.7)	1277 (5.3)	0.069	0.103	0.102	NA	NA
East Africa	312 (0.9)	937 (9.2)	677 (10.6)	0.390	0.430	4996 (2.3)	1599 (10.8)	3356 (14.0)	0.116	0.435	0.518	NA	NA
Southern Africa	344 (0.9)	120 (1.2)	47 (0.7)	0.024	0.022	916 (0.4)	8 (0.1)	75 (0.3)	0.063	0.126	0.08	NA	NA
Middle East	4157 (11.4)	7120 (70.2)	1455 (22.9)	1.494	0.309	15 456 (7.1)	3189 (21.5)	1547 (6.4)	0.148	0.277	0.173	NA	NA
North Africa	1056 (2.9)	482 (4.8)	360 (5.7)	0.097	0.137	3845 (1.8)	458 (3.1)	410 (1.7)	0.075	0.012	0.079	NA	NA
Central America	637-643	1-5	105 (1.7%)	0.180	0.007	3867 (1.8)	1099 (7.4)	960 (4.0)	0.002	0.274	0.135	NA	NA
South America	868 (2.4)	29 (0.3)	269 (4.2)	0.180	0.104	9915 (4.5)	336 (2.3)	1787 (7.4)	0.119	0.007	0.237	NA	NA
Caribbean	1000 (2.7)	1-5	141-146	0.230	0.029	13 006 (6.0)	20 (0.1)	1953 (8.1)	0.159	0.220	0.240	NA	NA
North America	3424 (9.4)	26 (0.3)	418 (6.6)	0.440	0.103	4496 (2.1)	87 (0.6)	113 (0.5)	0.318	0.412	0.420	NA	NA
East Asia	2793 (7.6)	6 (0.1)	302 (4.7)	0.400	0.120	31 159 (14.3)	60 (0.4)	2834 (11.8)	0.214	0.374	0.141	NA	NA
Australasia, Oceania, and Asia unspecified	270-275	0	1-5	0.120	0.108	572 (0.3)	9 (0.1)	8 (0.0)	0.069	0.109	0.115	NA	NA
Southeast Asia	2642 (7.2)	214 (2.1)	25 (0.4)	0.244	0.363	26 998 (12.4)	1168 (7.9)	256 (1.1)	0.174	0.025	0.313	NA	NA
South Asia	14 326 (39.2)	575 (5.7)	1030 (16.2)	0.877	0.531	66 084 (30.3)	3091 (20.9)	6081 (25.3)	0.187	0.407	0.299	NA	NA
Eastern Europe	1080 (3.0)	42 (0.4)	482 (7.6)	0.198	0.208	18 068 (8.3)	1738 (11.7)	1877 (7.8)	0.233	0.342	0.217	NA	NA
Europe other	2301 (6.3)	448 (4.4)	498 (7.8)	0.083	0.060	13 322 (6.1)	1387 (9.4)	714 (3.0)	0.008	0.115	0.158	NA	NA
Not stated or missing	0	0	0	NA	NA	0	1-5	1-5	NA	0.012	0.013	NA	NA
**Adolescents**													
Total, No.	75 665	14 085	15 225	NA	NA	192 512	11 542	17 927	NA	NA	NA	815 473	NA
Age, y													
Mean (SD)	14.32 (1.99)	13.99 (1.99)	14.30 (1.96)	0.170	0.012	13.86 (1.96)	13.91 (1.99)	13.77 (1.94)	0.236	0.208	0.281	14.01 (1.99)	0.159
11-14	37 861 (50.0)	8121 (57.7)	7785 (51.1)	0.153	0.022	116 838 (60.7)	6826 (59.1)	11 237 (62.7)	0.216	0.184	0.257	466 377 (57.2)	0.144
15-17	37 804 (50.0)	5964 (42.3)	7440 (48.9)	0.153	0.022	75 674 (39.3)	4716 (40.9)	6690 (37.3)	0.216	0.184	0.257	349 096 (42.8)	0.144
Sex													
Female	36 175 (47.8)	6862 (48.7)	7468 (49.1)	0.018	0.025	93 345 (48.5)	5652 (49.0)	8871 (49.5)	0.014	0.023	0.034	397 439 (48.7)	0.019
Male	39 490 (52.2)	7223 (51.3)	7757 (50.9)	0.018	0.025	99 167 (51.5)	5890 (51.0)	9056 (50.5)	0.014	0.023	0.034	418 034 (51.3)	0.019
Material deprivation quintile													
1 (Least deprived)	17 008 (22.5)	1146 (8.1)	1829 (12.0)	0.406	0.28	41 313 (21.5)	2296 (19.9)	2681 (15.0)	0.025	0.063	0.194	216 415 (26.5)	0.095
2	15 560 (20.6)	1604 (11.4)	1847 (12.1)	0.252	0.230	41 638 (21.6)	2117 (18.3)	2973 (16.6)	0.026	0.056	0.102	185 474 (22.7)	0.053
3	14 022 (18.5)	1988 (14.1)	2274 (14.9)	0.120	0.096	38 199 (19.8)	1944 (16.8)	3155 (17.6)	0.033	0.044	0.024	147 650 (18.1)	0.011
4	13 807 (18.2)	2826 (20.1)	3111 (20.4)	0.046	0.055	34 772 (18.1)	1855 (16.1)	3533 (19.7)	0.005	0.058	0.037	125 300 (15.4)	0.077
5 (Most deprived)	15 268 (20.2)	6521 (46.3)	6164 (40.5)	0.577	0.453	36 590 (19.0)	3330 (28.9)	5585 (31.2)	0.030	0.203	0.253	140 634 (17.2)	0.075
Region of origin													
Central Africa	253 (0.3)	285 (2.0)	314 (2.1)	0.157	0.159	351 (0.2)	140 (1.2)	564 (3.1)	0.030	0.100	0.216	NA	NA
Western Africa	1607 (2.1)	95 (0.7)	1061 (7.0)	0.124	0.234	3595 (1.9)	199 (1.7)	886 (4.9)	0.018	0.029	0.153	NA	NA
East Africa	882 (1.2)	1682 (11.9)	1407 (9.2)	0.446	0.370	4596 (2.4)	1342 (11.6)	3290 (18.4)	0.093	0.438	0.605	NA	NA
Southern Africa	388 (0.5)	96 (0.7)	105 (0.7)	0.022	0.023	826 (0.4)	10-15	9-14	0.012	0.072	0.082	NA	NA
Middle East	11 090 (14.7)	9266 (65.8)	2147 (14.1)	1.222	0.016	12 463 (6.5)	1447 (12.5)	1172 (6.5)	0.269	0.062	0.266	NA	NA
North Africa	2399 (3.2)	346 (2.5)	442 (2.9)	0.043	0.016	3201 (1.7)	494 (4.3)	390 (2.2)	0.098	0.059	0.062	NA	NA
Central America	837 (1.1)	12 (0.1)	457 (3.0)	0.133	0.134	3519 (1.8)	1272 (11.0)	546 (3.0)	0.060	0.425	0.136	NA	NA
South America	1772 (2.3)	123 (0.9)	598 (3.9)	0.117	0.091	9280 (4.8)	281 (2.4)	1231 (6.9)	0.134	0.006	0.217	NA	NA
Caribbean	3560 (4.7)	7 (0.0)	835 (5.5)	0.309	0.035	13 536 (7.0)	22 (0.2)	626 (3.5)	0.099	0.295	0.061	NA	NA
North America	5836 (7.7)	7 (0.0)	2153 (14.1)	0.405	0.207	3605 (1.9)	55-60	31-36	0.276	0.369	0.394	NA	NA
East Asia	5567 (7.4)	15 (0.1)	966 (6.3)	0.390	0.04	24 929 (12.9)	47 (0.4)	1685 (9.4)	0.186	0.366	0.074	NA	NA
Australasia, Oceania, and Asia unspecified	428 (0.6)	0	11 (0.1)	0.107	0.088	580 (0.3)	16-21	1-5	0.040	0.068	0.100	NA	NA
Southeast Asia	11 477 (15.2)	365 (2.6)	89 (0.6)	0.453	0.562	21 628 (11.2)	1098 (9.5)	199 (1.1)	0.116	0.173	0.532	NA	NA
South Asia	21 127 (27.9)	1334 (9.5)	2748 (18.0)	0.487	0.236	61 882 (32.1)	2088 (18.1)	5268 (29.4)	0.092	0.235	0.032	NA	NA
Eastern Europe	3654 (4.8)	262 (1.9)	1021 (6.7)	0.166	0.081	15 781 (8.2)	1713 (14.8)	1333 (7.4)	0.137	0.341	0.109	NA	NA
Europe other	4788 (6.3)	190 (1.3)	871 (5.7)	0.261	0.026	12 736 (6.6)	1307 (11.3)	687 (3.8)	0.012	0.177	0.114	NA	NA
Not stated or missing	0	0	0	NA	NA	1-5	1-5	0	0.006	0.019	NA	NA	NA

Abbrevation: NA, not applicable.

^a^ Small cells (<6) suppressed, and in the case of nonmissing data other cells may be reported as ranges without percentage to reduce risk of reidentification in accordance with ICES policy.

^b^ Immigrant-related characteristics of second generation immigrants and refugees are those of their mother or birthing parent.

^c^ Standardized differences compared with first-generation immigrants.

^d^ Immigrants include economic immigrants and sponsored family immigrants.

^e^ Resettled refugees include privately sponsored and government-assisted refugees.

^f^ Protected persons include successful asylum seekers and their dependents.

### COVID-19 Vaccine Coverage

A total of 583 160 children (53.1%) and 905 131 adolescents (79.2%) were vaccinated by study end (Table 2). Vaccine coverage was significantly different by immigration category. There were higher or similar rates of vaccination in immigrants compared with nonimmigrants (children: 60.2% vs 54.3%; adolescents: 79.1% vs 79.2%), but across age groups and generations, resettled refugees had lower vaccine coverage compared with immigrants and nonimmigrants (refugee children: 27.7%; refugee adolescents: 74.2%). Vaccination varied significantly by region of origin, with the lowest rates in immigrants and refugees from Eastern Europe (children: 20.6%; adolescents: 59.3%) and Central Africa (children: 20.6%; adolescents: 59.3%) and the highest rates in immigrants and refugees from Southeast Asia (children: 74.2%; adolescents: 92.8%). Vaccination rates increased with age (Table 2).

**Table 2.** Vaccine Coverage by Characteristics of First- and Second-Generation Immigrants and Refugees and All Nonimmigrant Children and Adolescents on April 24, 2022

Characteristic	Children (age 4-10 y)				Adolescents (age 11-17 y)				
	No. (row %)		Total, No	*P* value	No. (row %)			Total, No.	*P* value
	Unvaccinated	≥1 Dose			Unvaccinated	1 Dose	≥2 Doses		
Total, No.	515 589 (46.9)	583 160 (53.1)	1 098 749	NA	212 504 (18.6)	24 794 (2.2)	905 131 (79.2)	1 142 429	NA
Age, y									
4-7	310 950 (50.6)	303 076 (49.4)	614 026	<.001	NA	NA	NA	NA	<.001
8-10	204 639 (42.2)	280 084 (57.8)	484 723		NA	NA	NA	NA	
11-14	NA	NA	NA		134 338 (20.5)	15 416 (2.4)	505 291 (77.1)	655 045	
15-17	NA	NA	NA		78 166 (16.0)	9378 (1.9)	399 840 (82.0)	487 384	
Sex									
Female	250 568 (46.8)	284 793 (53.2)	535 361	.013	99 122 (17.8)	11 580 (2.1)	445 110 (80.1)	555 812	<.001
Male	265 021 (47)	298 367 (53)	563 388		113 382 (19.3)	13 214 (2.3)	460 021 (78.4)	586 617	
Immigration category									
First generation									
Immigrants^a^	14 545 (39.8)	22 039 (60.2)	36 584	<.001	14 521 (19.2)	1286 (1.7)	59 858 (79.1)	75 665	<.001
Resettled refugees^b^	7335 (72.3)	2809 (27.7)	10 144		2550 (18.1)	1086 (7.7)	10 449 (74.2)	14 085	
Protected persons and others^c^	4045 (63.6)	2317 (36.4)	6362		3049 (20.0)	656 (4.3)	11 520 (75.7)	15 225	
Second generation									
Immigrants	104 038 (47.7)	114 055 (52.3)	218 093		33 301 (17.3)	4017 (2.1)	155 194 (80.6)	192 512	
Resettled refugees	10 230 (69.1)	4570 (30.9)	14 800		2737 (23.7)	450 (3.9)	8355 (72.4)	11 542	
Protected persons and others	14 796 (61.7)	9197 (38.3)	23 993		3563 (19.9)	613 (3.4)	13 751 (76.7)	17 927	
Nonimmigrants	360 600 (45.7)	428 173 (54.3)	788 773		152 783 (18.7)	16 686 (2.0)	646 004 (79.2)	815 473	
Recency of immigration^d^									
Recent (0 to ≤5 y)	17 833 (51.9)	16 529 (48.1)	34 362	<.001	4456 (15.3)	1426 (4.9)	23 150 (79.7)	29 032	<.001
Intermediate (>5 to ≤10 y)	7962 (43.3)	10 439 (56.7)	18 401		7227 (19.0)	991 (2.6)	29 899 (78.4)	38 117	
Long-term (>10 y)	130 (39.8)	197 (60.2)	327		8437 (22.3)	611 (1.6)	28 778 (76.1)	37 826	
Region of origin^e^									
Central Africa	1728 (80.5)	418 (19.5)	2146	<.001	607 (31.8)	123 (6.4)	1177 (61.7)	1907	<.001
Western Africa	4,793 (60.9)	3,074 (39.1)	7867		1506 (20.2)	326 (4.4)	5611 (75.4)	7443	
East Africa	8421 (70.9)	3456 (29.1)	11 877		3004 (22.8)	735 (5.6)	9460 (71.7)	13 199	
Southern Africa	617 (40.9)	893 (59.1)	1510		274 (19.0)	34 (2.4)	1133 (78.6)	1441	
Middle East	20 999 (63.8)	11 925 (36.2)	32 924		8067 (21.5)	1454 (3.9)	28 064 (74.7)	37 585	
North Africa	4423 (66.9)	2188 (33.1)	6611		1583 (21.8)	208 (2.9)	5481 (75.4)	7272	
Central America	3796 (56.9)	2878 (43.1)	6674		1629 (24.5)	129 (1.9)	4885 (73.5)	6643	
South America	6080 (46.0)	7124 (54.0)	13 204		2048 (15.4)	261 (2.0)	10 976 (82.6)	13 285	
Caribbean	11 466 (71.1)	4662 (28.9)	16 128		5988 (32.2)	717 (3.9)	11 881 (63.9)	18 586	
North America	3969 (46.3)	4595 (53.7)	8564		3446 (29.5)	226 (1.9)	8021 (68.6)	11 693	
East Asia	11 635 (31.3)	25 519 (68.7)	37 154		4061 (12.2)	385 (1.2)	28 763 (86.6)	33 209	
Australasia, Oceania, and Asia unspecified	366 (42.2)	501 (57.8)	867		252 (24.2)	18 (1.7)	771 (74.1)	1041	
Southeast Asia	9388 (30.0)	21 915 (70.0)	31 303		2455 (7.0)	400 (1.1)	32 001 (91.8)	34 856	
South Asia	39 077 (42.9)	52 110 (57.1)	91 187		10 893 (11.5)	2020 (2.1)	81 534 (86.3)	94 447	
Eastern Europe	17 198 (73.9)	6089 (26.1)	23 287		8585 (36.1)	605 (2.5)	14 574 (61.3)	23 764	
Europe other	11 030 (59.1)	7640 (40.9)	18 670		5323 (25.9)	467 (2.3)	14 789 (71.9)	20 579	
Rural residence									
Yes	60 085 (53.2)	52 867 (46.8)	112 952	<.001	26 523 (23.9)	2946 (2.7)	81 665 (73.5)	111 134	<.001
No	454 263 (46.2)	529 554 (53.8)	983 817		185 133 (18.0)	21 797 (2.1)	822 003 (79.9)	1 028 933	
Missing	1241 (62.7)	739 (37.3)	1980		848 (35.9)	51 (2.2)	1463 (61.9)	2362	
Material deprivation quintile									
1 (Least deprived)	97 066 (36.2)	170 795 (63.8)	267 861	<.001	37 674 (13.3)	3722 (1.3)	241 292 (85.4)	282 688	<.001
2	98 909 (41.9)	136 945 (58.1)	235 854		39 167 (15.6)	4083 (1.6)	207 963 (82.8)	251 213	
3	94 853 (47.7)	103 893 (52.3)	198 746		38 614 (18.5)	4306 (2.1)	166 312 (79.5)	209 232	
4	95 790 (52.7)	85 905 (47.3)	181 695		39 647 (21.4)	4761 (2.6)	140 796 (76.0)	185 204	
5 (Most deprived)	128 971 (60.1)	85 622 (39.9)	214 593		57 402 (26.8)	7922 (3.7)	148 768 (69.5)	214 092	
Neighborhood COVID-19 risk decile^e^									
1 (Most at risk)	69 524 (56.7)	53 025 (43.3)	122 549	<.001	27 605 (21.3)	3648 (2.8)	98 639 (75.9)	129 892	<.001
2	54 538 (49.6)	55 379 (50.4)	109 917		22 651 (19.1)	2572 (2.2)	93 590 (78.8)	118 813	
3	54 925 (50.1)	54 680 (49.9)	109 605		22 421 (19.6)	2497 (2.2)	89 584 (78.2)	114 502	
4	53 666 (49.5)	54 803 (50.5)	108 469		22 828 (20.4)	2605 (2.3)	86 648 (77.3)	112 081	
5	49 630 (44.6)	61 634 (55.4)	111 264		19 592 (17.2)	2229 (2.0)	92 006 (80.8)	113 827	
6	48 649 (44.2)	61 523 (55.8)	110 172		21 061 (18.3)	2291 (2.0)	91 855 (79.7)	115 207	
7	42 211 (41.2)	60 264 (58.8)	102 475		18 291 (17.2)	1801 (1.7)	86 552 (81.2)	106 644	
8	45 749 (41.0)	65 759 (59.0)	111 508		18 735 (16.0)	1935 (1.7)	96 547 (82.4)	117 217	
9	48 671 (44.0)	61 956 (56.0)	110 627		19 657 (17.4)	2542 (2.3)	90 547 (80.3)	112 746	
10 (Least at risk)	48 009 (47.0)	54 122 (53.0)	102 131		19 658 (19.4)	2674 (2.6)	79 137 (78.0)	101 469	
Previous SARS-CoV-2 infection									
No	499 210 (46.8)	567 582 (53.2)	1 066 792	<.001	208 950 (18.8)	23 917 (2.2)	878 113 (79.0)	1 110 980	<.001
Yes	16 379 (51.3)	15 578 (48.7)	31 957		3554 (11.3)	877 (2.8)	27 018 (85.9)	31 449	
Has a pediatric chronic condition									
No	485 254 (47.0)	547 905 (53.0)	1 033 159	<.001	173 027 (19.8)	19 063 (2.2)	680 578 (78.0)	872 668	<.001
Yes	30 335 (46.2)	35 255 (53.8)	65 590		39 477 (14.6)	5731 (2.1)	224 553 (83.2)	269 761	
Primary care access model									
Community health center	12 750 (53.9)	10 887 (46.1)	23 637	<.001	4390 (18.8)	902 (3.9)	18 063 (77.3)	23 355	<.001
Rostered to a primary health care practitioner	319 888 (44.2)	404 306 (55.8)	724 194		127 281 (15.3)	16 978 (2.0)	687 209 (82.7)	831 468	
Pediatrician	28 786 (36.6)	49 909 (63.4)	78 695		4982 (11.0)	660 (1.5)	39 610 (87.5)	45 252	
Noncomprehensive care	81 999 (50.2)	81 229 (49.8)	163 228		20 008 (16.3)	3500 (2.9)	99 103 (80.8)	122 611	
No regular health care practitioner	72 166 (66.2)	36 829 (33.8)	108 995		55 843 (46.6)	2754 (2.3)	61 146 (51.1)	119 743	
Influenza vaccination in 2019-2020									
No	486 412 (50.1)	484 753 (49.9)	971 165	<.001	209 465 (19.3)	24 004 (2.2)	849 460 (78.4)	1 082 929	<.001
Yes	29 177 (22.9)	98 407 (77.1)	127 584		3039 (5.1)	790 (1.3)	55 671 (93.6)	59 500	

Abbreviation: NA, not applicable or not available.

^a^ Immigrants include economic immigrants and sponsored family immigrants.

^b^ Resettled refugees include privately sponsored and government-sponsored refugees.

^c^ Protected persons include successful asylum seekers and their dependents.

^d^ Recency is only reported for first-generation immigrants and refugees.

^e^ Vaccination rates not reported in missing categories with small cells.

### Vaccination in Immigrants and Refugees and by Generation

**Figure 1.**
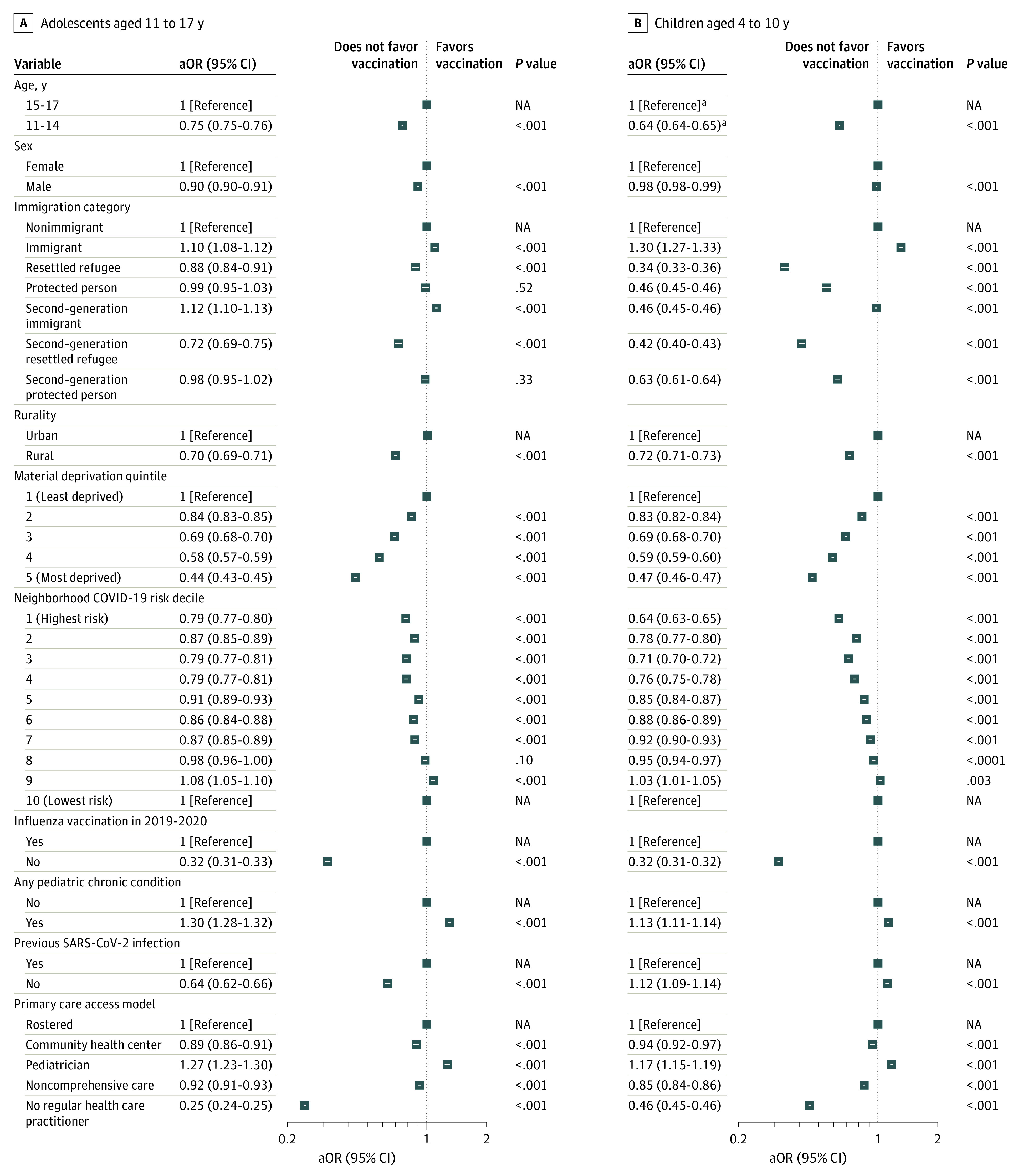
Adjusted Odds Ratios (aORs) for Being Vaccinated Among Children and Adolescents in Ontario, Canada, by April 24, 2022 Children were considered vaccinated if they had received at least 1 vaccine dose; adolescents, at least 2 vaccine doses. NA indicates not applicable. ^a^For children, the reference age group was 8 to 10 years, and data are given for children aged 4 to 7 years.

Compared with nonimmigrants, immigrants had higher odds of being vaccinated, an association that persisted after adjustment (children: aOR, 1.30; 95% CI, 1.27-1.33; adolescents: aOR, 1.10; 95% CI, 1.08-1.12) (Figure 1) and for immigrant adolescents across generation. Conversely, resettled refugees had lower odds of being vaccinated compared with nonimmigrants (children: aOR, 0.34; 95% CI, 0.33-0.36; adolescents: aOR, 0.88; 95% CI, 0.84-0.91). Odds for protected-person children were lower (aOR, 0.55; 95% CI, 0.52-0.58), whereas adolescent protected persons had similar odds of uptake compared with nonimmigrants (aOR, 0.99; 95% CI, 0.95-1.03). The lower odds of vaccination in resettled refugees persisted across generations for both cohorts, whereas there was an attenuation of difference in the odds in second-generation immigrant children (aOR, 0.98; 95% CI, 0.97-0.99) (eTable 5 in Supplement 1). Vaccination coverage was lower with increasing material deprivation for all minors, but the association with neighborhood COVID-19 risk decile was less consistent, particularly in adolescents (Figure 1). Individuals with chronic conditions had higher odds of vaccination compared with healthy peers, especially among adolescents (children: aOR, 1.13; 95% CI, 1.11-1.14; adolescents: aOR, 1.30; 95% CI, 1.28-1.32). While having no primary health care practitioner was strongly associated with lower odds of vaccination, children and adolescents with a pediatrician had higher odds of vaccination (Figure 1).

**Figure 2.**
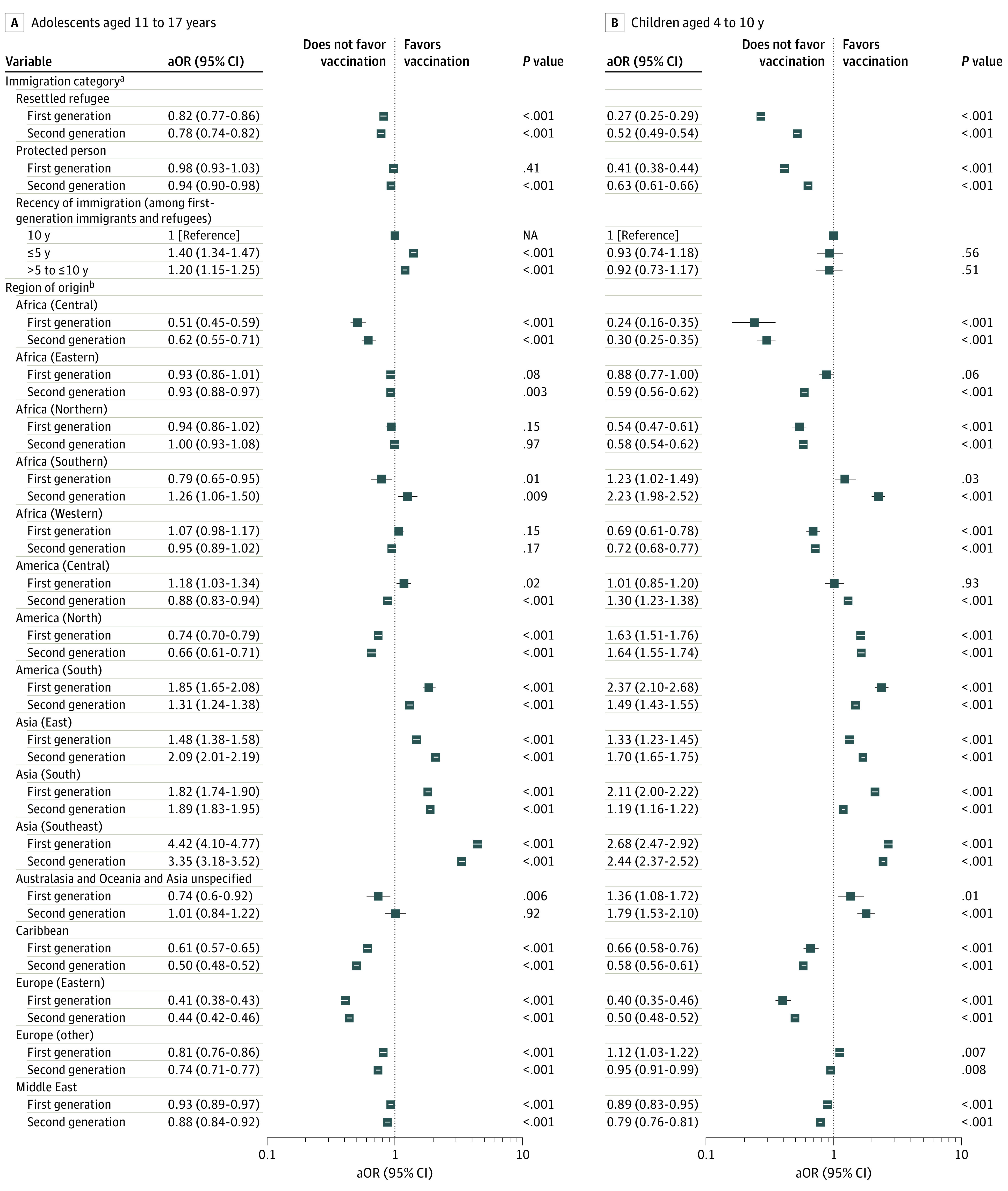
Adjusted Odds Ratios (aORs) for Being Vaccinated Among First- and Second-Generation Immigrant Children and Adolescents in Ontario, Canada, by April 24, 2022 Children were considered vaccinated if they had received at least 1 vaccine dose; adolescents, at least 2 vaccine doses. NA indicates not applicable. ^a^First-generation immigrants are the reference group for other first-generation individuals; second-generation immigrants, other second-generation individuals. ^b^The full cohort of first-generation immigrants and refugees are the reference group for first-generation immigrants and refugees by region of origin; the full cohort of second-generation immigrants and refugees, second-generation immigrants and refugees by region of origin.

The analysis by immigrant or refugee generation showed a strong association between vaccination and region of origin after adjustment for other sociodemographic factors. Compared with the overall vaccination rate for all immigrants and refugees, odds were lowest for children and adolescents from Eastern Europe (children: aOR, 0.40; 95% CI, 0.35-0.46; adolescents: aOR, 0.41; 95% CI, 0.38-0.43) and Central Africa (children: aOR, 0.24; 95% CI, 0.16-0.35; adolescents: aOR, 0.51; 95% CI, 0.45-0.59) and highest for children and adolescents from Southeast Asia (children: aOR, 2.68; 95% CI, 2.47-2.92; adolescents: aOR, 4.42; 95% CI, 4.10-4.77) (Figure 2; eTable 6 and eTable 7 in Supplement 1). For almost all regions of origin with low vaccination rates, the adjusted odds were similarly low in the second generation. Socioeconomic inequities were present in the immigrant- and refugee-only model but less pronounced than in the model including all Ontario minors (eTables 5-7 in Supplement 1).

### Time to First Vaccination

The time-to-event analyses showed similar patterns to the logistic regression models (Table 3). There was earlier uptake of the first COVID-19 vaccine dose in immigrants compared with nonimmigrants (adjusted HR, 1.05; 95% CI, 1.04-1.06). In the immigrant- and refugee-only model, wide ranges in the time to vaccination existed among different regions of origin, with faster uptake seen in regions with higher vaccine coverage. An exception was observed among children and adolescents from South America, with a relatively slow vaccine uptake (adjusted HR, 0.69; 95% CI, 0.68-0.71) paired with an vaccination rate higher than the overall rate (immigrant children: aOR, 2.37; 95% CI, 2.10-2.68) (Table 3; eTable 6 in Supplement 1).

**Table 3.** Hazard of First Vaccination in Full Cohort and Immigrants and Refugees Among Adolescents and Children on April 24, 2022

Parameter	Adjusted HR (95% CI)	
	Full cohort (N = 2 236 799)	First and second generation immigrants/refugees (n = 635 868)
Age group, y		
4-7	0.39 (0.39-0.39)	0.30 (0.30-0.30)
8-10	0.53 (0.52-0.53)	0.40 (0.40-0.40)
11-14	0.80 (0.79-0.80)	0.76 (0.76-0.77)
15-17	1 [Reference]	1 [Reference]
Male sex (vs female)	0.96 (0.95-0.96)	0.96 (0.95-0.96)
Immigration category		
Nonimmigrants	1 [Reference]	NA
First generation		
Immigrants^a^	1.05 (1.04-1.06)	1 [Reference]
Resettled refugees^b^	0.67 (0.66-0.68)	0.72 (0.71-0.74)
Protected persons and others^c^	0.82 (0.80-0.83)	0.85 (0.83-0.87)
Second generation		
Immigrants^a^	0.96 (0.96-0.97)	0.95 (0.94-0.96)
Resettled refugees^b^	0.63 (0.62-0.64)	0.68 (0.67-0.70)
Protected persons and others^c^	0.77 (0.76-0.78)	0.82 (0.81-0.83)
Recency of immigration		
Recent (0 to ≤5 y)	NA	1.04 (1.03-1.06)
Intermediate (>5 to ≤10 y)	NA	0.97 (0.96-0.98)
Long-term (>10 y)	NA	1 [Reference]
Region of origin (Ref Southeast Asia)		
Australasia & Oceania & Asia unspecified	NA	0.67 (0.64-0.71)
Caribbean	NA	0.35 (0.35-0.36)
Central Africa	NA	0.33 (0.32-0.35)
Central America	NA	0.57 (0.56-0.59)
East Africa	NA	0.5 (0.49-0.51)
East Asia	NA	0.91 (0.90-0.92)
Eastern Europe	NA	0.32 (0.32-0.33)
Europe other	NA	0.50 (0.50-0.51)
Middle East	NA	0.51 (0.50-0.52)
North Africa	NA	0.49 (0.48-0.50)
North America	NA	0.57 (0.56-0.58)
South America	NA	0.69 (0.68-0.71)
Southeast Asia	NA	1 [Reference]
South Asia	NA	0.80 (0.79-0.81)
Southern Africa	NA	0.76 (0.73-0.80)
Western Africa	NA	0.48 (0.47-0.49)
Rural (vs urban)	0.80 (0.80-0.81)	0.71 (0.68-0.73)
Material Deprivation Quintile		
1 (Least deprived)	1 [Reference]	1 [Reference]
2	0.91 (0.90-0.91)	0.96 (0.95-0.97)
3	0.81 (0.81-0.81)	0.88 (0.87-0.89)
4	0.73 (0.72-0.73)	0.83 (0.82-0.84)
5 (Most deprived)	0.61 (0.60-0.61)	0.76 (0.75-0.77)
Neighborhood COVID-19 risk decile		
1 (Most at risk)	0.92 (0.91-0.92)	0.88 (0.85-0.90)
2	1.00 (0.99-1.01)	0.92 (0.90-0.95)
3	0.92 (0.91-0.92)	0.84 (0.82-0.87)
4	0.93 (0.92-0.93)	0.85 (0.83-0.88)
5	0.99 (0.98-0.99)	0.89 (0.87-0.92)
6	1.00 (0.99-1.00)	0.89 (0.86-0.91)
7	0.99 (0.98-1.00)	0.88 (0.86-0.90)
8	1.01 (1.00-1.02)	0.90 (0.88-0.93)
9	0.99 (0.99-1.00)	0.86 (0.83-0.89)
10 (Least at risk)	1 [Reference]	1 [Reference]
No influenza vaccination in 2019-2020	0.54 (0.54-0.54)	0.64 (0.63-0.65)
Has a pediatric chronic condition	1.10 (1.09-1.10)	1.11 (1.10-1.12)
Primary care access model		
Rostered	1 [Reference]	1 [Reference]
Community health center	0.94 (0.93-0.95)	1.01 (0.99-1.03)
Pediatrician	1.15 (1.14-1.16)	1.10 (1.08-1.11)
Noncomprehensive care	0.92 (0.92-0.93)	0.99 (0.98-1.00)
No regular care practitioner	0.49 (0.49-0.49)	0.43 (0.43-0.44)
No previous COVID-19 infection	1.00 (0.99-1.01)	0.91 (0.90-0.93)

Abbreviation: NA, not applicable or not available.

^a^ Immigrants include economic immigrants and sponsored family immigrants.

^b^ Resettled refugees include privately sponsored and government sponsored refugees.

^c^ Protected persons and others include successful asylum seekers and their dependents.

## Discussion

In this population-based cohort study, COVID-19 vaccination coverage was 53.1% in children and 79.2% in adolescents in Ontario, Canada. Vaccination rates were higher for immigrants and lower for refugees, compared with nonimmigrants. There was significant variation within subgroups of immigrants and refugees by region of origin, with relative differences frequently persisting across generations. Odds of vaccination increased with age, higher socioeconomic status, and lower neighborhood COVID-19 risk. Similar associations were found when analyzing time to first vaccination.

Vaccine acceptance is complex,^46^ as recently summarized in the 5C model: besides vaccine confidence, complacency (not perceiving diseases as high risk), constraints (structural or psychological barriers), calculation (engagement in extensive information searching), and collective responsibility were identified as important factors associated with vaccine acceptance.^47,48^ For migrants, a systematic review on routine and COVID-19 vaccination named language, vaccine literacy, and vaccination benefits as additional factors.^49^ All of these factors likely contributed to our results.

Our findings of higher vaccine coverage in immigrants is consistent with a study of adolescents and adults from Alberta, Canada, which found higher vaccine coverage (78% vs 76%) in immigrants compared with nonimmigrants. However, it did not distinguish immigrants and refugees, assess generations, or include children.^50^ In our study, refugees, and particularly refugee children, were more likely to be undervaccinated, which is consistent with data on routine and COVID-19 immunizations in European refugees.^10^ While potential explanations include different countries of origin or socioeconomic status in refugees compared with other immigrants, we adjusted for both, suggesting an independent association between refugee status and undervaccination. Educational attainment is higher in protected persons than in resettled refugees^51^ and may explain differences within refugee groups in our study. Although evidence on parental education and vaccination acceptance is not universal,^52,53,54^ studies from North America, New Zealand, and Europe have reported a positive association.^55,56,57,58,59^ Other explanations may be limited language ability or health literacy and lower confidence in vaccine information from the media.^59^

We found a high degree of variation by region of origin (the range of differences in rates was 29.5 percentage points for second generation immigrant and refugee adolescents and 53.6 percentage points for immigrant and refugee children). Associations between regions of origin and vaccination were not attenuated after adjustment for socioeconomic and demographic factors. Low rates in immigrants and refugees from Eastern Europe and Central Africa were consistent with other literature,^16^ and low uptake has also been documented for citizens in the corresponding home countries.^60^ While cross-national frameworks for research on immigrant health are complex and causes for health behaviors highly context-specific,^60,61^ low vaccine coverage in Eastern European immigrants and refugees is well documented.^62,63,64^ A study on routine vaccine hesitancy in Eastern Europe elucidated potential factors, including conspiracy theories and reduced confidence in medical science and institutions, particularly if combined with low objective vaccine knowledge^65^; these factors likely to contribute to undervaccination observed in Eastern European immigrants in our study. However for Central African immigrants, structural racism^66^ and systemic discrimination against Black individuals in Canada might have catalyzed the spread of disinformation and mediated undervaccination, as described in a 2023 qualitative study by Kemei et al.^67^ Distinguishing factors associated with vaccine hesitancy can help to better tailor public health campaigns.^65^ A 2021 study by Ganczak et al^68^ found Ukrainian refugees were more willing to receive COVID-19 vaccinations in countries other than their own, considering the host country’s health system more trustworthy. For Black individuals in Canada, strategies like town-hall events organized by the Black Scientists’ Task Force on Vaccine Equity were associated with successfully decreasing vaccine hesitancy.^28^ Our finding of high vaccine coverage in immigrants and refugees from Southeast Asia correspond with current evidence^60^ describing high confidence in vaccinations and health experts in these regions.^69^ Contrary to our hypotheses, associations by region of origin, especially in regions with low vaccination, were remarkably stable across generations, suggesting that cultural background influences vaccination decisions over longer periods of time than expected. While there is little comparable literature, this is consistent with a 2019 US study,^70^ in which self-reported influenza vaccine coverage was similar across first- and second-generation Arab immigrants.

We found lower material deprivation was associated with higher vaccination coverage. This is consistent with a systematic review by Wang et al^71^ reporting higher adult COVID-19 vaccination rates in higher-income households. A 2021 Canadian survey study^19^ described similar disparities, with lower-income parents being less likely to accept COVID-19 vaccinations for their children. The negative association of COVID-19 neighborhood risk decile and vaccination was less pronounced in adolescents compared with children, likely reflecting a mitigating effect of the adolescent COVID-19 vaccine campaign that specifically targeted high-risk neighborhoods.^72^ The undervaccination of individuals without a primary health care practitioner aligns with a 2022 Canadian mixed-methods analysis by Kholina et al^73^ describing primary care practitioners as key drivers of vaccine uptake, highlighting the importance of primary care for vaccine equity.

Other findings of our study correlate with survey data. Vaccination was higher with each incremental age increase, which was anticipated by parental surveys from Asia, the Middle East, North America, and Europe.^74,75,76,77,78,79^ Lower perceived risk of developing severe COVID-19, combined with less confidence in the relatively new COVID-19 vaccine for younger children, were the main reasons reported for vaccine hesitancy.^74,75,76,77,78,79^ Lower effectiveness of COVID-19 vaccinations in younger age groups may also contribute to reduced vaccine confidence.^79,80^

### Limitations

This study has some limitations. The use of administrative data and the retrospective design limited our ability to measure potentially important variables, including individual household income, parent or guardian education, language preference and proficiency, routine vaccine uptake, sources of information about COVID-19, peer group influence, and trust in the health system. As testing criteria for SARS-CoV-2 infections changed over time, this variable was limited to infections prior to vaccine eligibility. We had no data on immigrants or refugees who intended to arrive in another province, were undocumented migrants, or were asylum seekers awaiting their hearings, limiting generalizability of our findings to these groups. Additionally, the research took place in a context where publicly funded, equity-focused COVID-19 vaccination campaigns existed, where pediatric vaccination was recommended and promoted, and with distinct immigration policies. While resettled refugees in Canada may be similar to those in other countries, protected persons and immigrants may have different attributes than in other jurisdictions.

## Conclusions

In this Canadian population-based cohort study, nonrefugee immigrant minors had higher vaccine coverage than nonimmigrants. The substantial heterogeneity by region of origin and lower vaccination coverage in refugees persisted across generations. Precision public health approaches should target specific barriers in the identified, undervaccinated subgroups in ongoing vaccine campaigns.

## Notes

Supplement 1. **eTable 1.** List of Databases Used in the Study **eTable 2.** List of Variables Used in the Study **eTable 3.** Relevant Immigration Pathways in Canada and Context-Specific Definitions
eReferences.
**eTable 4.** Additional Baseline Characteristics of Immigrants, Refugees, Second-Generation Immigrants, Refugees, and All Nonimmigrant Children and Adolescents in Ontario on January 1, 2021 **eTable 5.** Crude and Adjusted Odds Ratios of Being Vaccinated (Full Cohorts) on April 24, 2022 **eTable 6.** Adjusted Odds Ratios of Being Vaccinated Among First- and Second-Generation Immigrant and Refugee Children on April 24, 2022 **eTable 7.** Adjusted Odds Ratios of Being Vaccinated Among First- and Second-Generation Immigrant and Refugee Adolescents on April 24, 2022 **eFigure 1.** Study Population Inclusion Flowchart **eFigure 2.** Milestones of the Ontario Vaccination Campaign in Relation to the Study Timeline

Supplement 2.
Data Sharing Statement

## References

[1] Flaxman S, Whittaker C, Semenova E, . Assessment of COVID-19 as the underlying cause of death among children and young people aged 0 to 19 years in the US. JAMA Netw Open. 2023;6(1):e2253590. doi:10.1001/jamanetworkopen.2022.53590 36716029 PMC9887489

[2] Johnson MS, Skjerdingstad N, Ebrahimi OV, Hoffart A, Urnes Johnson S. Mechanisms of parental distress during and after the first COVID-19 lockdown phase: a two-wave longitudinal study. PLoS One. 2021;16(6):e0253087. doi:10.1371/journal.pone.0253087 34166429 PMC8224894

[3] Kutsar D, Kurvet-Käosaar L. The impact of the COVID-19 pandemic on families: young people’s experiences in Estonia. Front Sociol. 2021;6. doi:10.3389/fsoc.2021.732984 PMC8425282 34513980

[4] Nowrouzi-Kia B, Osipenko L, Eftekhar P, . The early impact of the global lockdown on post-secondary students and staff: a global, descriptive study. SAGE Open Med. Published online January 25, 2022. doi:10.1177/20503121221074480 35096392 PMC8793123

[5] Zheng YJ, Wang XC, Feng LZ, ; China National Clinical Research Center for Respiratory Diseases; National Center for Children’s Health, Beijing, China; Group of Respirology, Chinese Pediatric Society, Chinese Medical Association; Chinese Medical Doctor Association Committee on Respirology Pediatrics; China Medicine Education Association Committee on Pediatrics; Chinese Research Hospital Association Committee on Pediatrics; Chinese Non-government Medical Institutions Association Committee on Pediatrics; China Association of Traditional Chinese Medicine, Committee on Children’s Health and Medicine Research. Expert consensus on COVID-19 vaccination in children. World J Pediatr. 2021;17(5):449-457. doi:10.1007/s12519-021-00465-6 34618327 PMC8494629

[6] Schleiss MR, John CC, Permar SR. Children are the key to the endgame: a case for routine pediatric COVID vaccination. Vaccine. 2021;39(38):5333-5336. doi:10.1016/j.vaccine.2021.08.005 34393021 PMC8358829

[7] Gerber JS, Offit PA. COVID-19 vaccines for children. Science. 2021;374(6570):913. doi:10.1126/science.abn2566 34793207

[8] Cauchemez S, Bosetti P, Kiem CT, Mouro V, Consoli A, Fontanet A. Education and mental health: good reasons to vaccinate children. Lancet. 2021;398(10298):387. doi:10.1016/S0140-6736(21)01453-7 34273293 PMC8279961

[9] Paul S, Mishra CM. Do we need to vaccinate every child against COVID-19: what evidence suggests—a systematic review of opinions. Front Public Health. 2022;10:1002992. doi:10.3389/fpubh.2022.1002992 36424958 PMC9679503

[10] Pannaraj PS. Immunization schedule updated for 2023; COVID vaccine added. *AAP News*. February 9, 2023. Accessed June 20, 2023. https://publications.aap.org/aapnews/news/23293/Immunization-schedule-updated-for-2023-COVID

[11] Government of Canada. Vaccines for children: COVID-19. Accessed June 20, 2023. https://www.canada.ca/en/public-health/services/vaccination-children/covid-19.html

[12] Goldman RD, Bone JN, Gelernter R, . National COVID-19 vaccine program progress and parents’ willingness to vaccinate their children. Hum Vaccin Immunother. 2021;17(12):4889-4895. doi:10.1080/21645515.2021.1999144 34797754 PMC8903906

[13] Chen F, He Y, Shi Y. Parents’ and guardians’ willingness to vaccinate their children against COVID-19: a systematic review and meta-analysis. Vaccines (Basel). 2022;10(2):179. doi:10.3390/vaccines10020179 35214638 PMC8880569

[14] Viswanath K, Bekalu M, Dhawan D, Pinnamaneni R, Lang J, McLoud R. Individual and social determinants of COVID-19 vaccine uptake. BMC Public Health. 2021;21(1):818. doi:10.1186/s12889-021-10862-1 33910558 PMC8081000

[15] Galanis P, Vraka I, Siskou O, Konstantakopoulou O, Katsiroumpa A, Kaitelidou D. Willingness, refusal and influential factors of parents to vaccinate their children against the COVID-19: a systematic review and meta-analysis. Prev Med. 2022;157:106994. doi:10.1016/j.ypmed.2022.106994 35183597 PMC8861629

[16] Crawshaw AF, Farah Y, Deal A, . Defining the determinants of vaccine uptake and undervaccination in migrant populations in Europe to improve routine and COVID-19 vaccine uptake: a systematic review. Lancet Infect Dis. 2022;22(9):e254-e266. doi:10.1016/S1473-3099(22)00066-4 35429463 PMC9007555

[17] Schilling S, Orr CJ, Delamater AM, . COVID-19 vaccine hesitancy among low-income, racially and ethnically diverse US parents. Patient Educ Couns. 2022;105(8):2771-2777. doi:10.1016/j.pec.2022.03.023 35393230 PMC8966372

[18] Rane MS, Robertson MM, Westmoreland DA, Teasdale CA, Grov C, Nash D. Intention to vaccinate children against COVID-19 among vaccinated and unvaccinated US parents. JAMA Pediatr. 2022;176(2):201-203. doi:10.1001/jamapediatrics.2021.5153 34870702 PMC8649908

[19] McKinnon B, Quach C, Dubé È, Tuong Nguyen C, Zinszer K. Social inequalities in COVID-19 vaccine acceptance and uptake for children and adolescents in Montreal, Canada. Vaccine. 2021;39(49):7140-7145. doi:10.1016/j.vaccine.2021.10.077 34763947 PMC8573666

[20] Kaur U, Anju K, Chauhan M, . A prospective observational study on BBV152 coronavirus vaccine use in adolescents and comparison with adults- first real-world safety analysis. medRxiv. Preprint posted online April 10, 2022. doi:10.1101/2022.04.08.22273634 PMC9419918 36030299

[21] Government of Canada. Health Canada authorizes use of the Pfizer-BioNTech COVID-19 vaccine in children 12 to 15 years of age. News release. May 5, 2021. Accessed June 20, 2023. https://www.canada.ca/en/health-canada/news/2021/05/health-canada-authorizes-use-of-the-pfizer-biontech-covid-19-vaccine-in-children-12-to-15-years-of-age.html

[22] Government of Canada. Health Canada authorizes use of Comirnaty (the Pfizer-BioNTech COVID-19 vaccine) in children 5 to 11 years of age. News release. November 19, 2021. Accessed June 20, 2023. https://www.canada.ca/en/health-canada/news/2021/11/health-canada-authorizes-use-of-comirnaty-the-pfizer-biontech-covid-19-vaccine-in-children-5-to-11-years-of-age.html

[23] Government of Ontario. COVID-19 vaccine booking expanding to youth 12+ ahead of schedule. News release. May 21, 2021. Accessed June 20, 2023. https://news.ontario.ca/en/release/1000185/covid-19-vaccine-booking-expanding-to-youth-12-ahead-of-schedule

[24] Government of Ontario. Updated: executive officer notice: administration of publicly funded COVID 19 vaccines in Ontario pharmacies—eligibility. Accessed June 20, 2023. https://www.health.gov.on.ca/en/pro/programs/drugs/opdp_eo/notices/exec_office_eligibility_20211125.pdf

[25] Duchen R, Iskander C, Chung H, . The role of a resilient information infrastructure in COVID-19 vaccine uptake in Ontario. Healthc Q. 2021;24(2):7-11. doi:10.12927/hcq.2021.26553 34297657

[26] Public Health Ontario. COVID-19 in Ontario – a focus on neighbourhood diversity, February 26, 2020 to December 13, 2021. Accessed June 20, 2023. https://www.publichealthontario.ca/-/media/documents/ncov/epi/2020/06/covid-19-epi-diversity.pdf

[27] Office of the Auditor General of Ontario. Value-for-money audit: COVID-19 vaccination program. Accessed June 20, 2023. https://www.auditor.on.ca/en/content/annualreports/arreports/en22/AR_COVIDVaccination_en22.pdf

[28] Black Scientists’ Task Force on Vaccine Equity. Toronto’s Black community town halls unpacked. Accessed June 20, 2023. https://www.torontoblackcovid.com/assets/toronto-s-black-scientists-on-vaccine-equity-report-june-2021.pdf

[29] Taylor and Newberry Consultant. Evaluation of vaccine engagement teams: final report (executive summary). Accessed June 20, 2023. https://www.toronto.ca/wp-content/uploads/2022/09/8de1-VET-Final-Evaluation-Report-July-2022exec-summaryfinal.pdf

[30] Raza A. How Brampton went from a COVID-19 hotspot to one of Canada’s most vaccinated communities. *CBC*. November 30, 2021. Accessed June 20, 2023. https://www.cbc.ca/news/canada/toronto/brampton-covid-high-vaccinations-success-1.6264820

[31] Barrett KA, Feldman J, Trent J, ; the Ontario COVID-19 Science Advisory Table. COVID-19 vaccine confidence in Ontario and strategies to support capability, opportunity, and motivation among at risk populations, version 1.0. Accessed June 20, 2023. https://covid19-sciencetable.ca/sciencebrief/covid-19-vaccine-confidence-in-ontario-and-strategies-to-support-capability-opportunity-and-motivation-among-at-risk-populations/

[32] Warren M, Wallace K. Vaccination trend for Ontario kids 5-11 ‘alarming.’ *Toronto Star*. January 8, 2022. Accessed June 20, 2023. https://www.thestar.com/news/gta/2022/01/08/vaccination-trend-for-ontario-kids-5-11-alarming.html

[33] Government of Ontario. Indigenous peoples in Ontario. Accessed June 20, 2023. https://www.ontario.ca/document/spirit-reconciliation-ministry-indigenous-relations-and-reconciliation-first-10-years/indigenous-peoples-ontario#:~:text=Indigenous%20people%20represent%202.8%20per%20cent%20of%20the%20total%20population%20of%20Ontario

[34] Statistics Canada. Visible minority by immigrant status and period of immigration: Canada, provinces and territories, census metropolitan areas and census agglomerations with parts. Accessed June 20, 2023. https://www150.statcan.gc.ca/t1/tbl1/en/tv.action?pid=9810030801&pickMembers%5B0%5D=1.1&pickMembers%5B1%5D=2.1&pickMembers%5B2%5D=3.1&pickMembers%5B3%5D=4.1

[35] Glazier RH, Hutchison B, Kopp A. Comparison of Family Health Teams to Other Ontario Primary Care Models, 2004/05 to 2011/12. Accessed June 20, 2023. https://www.ices.on.ca/publications/research-reports/comparison-of-family-health-teams-to-other-primary-care-models-2004-05-to-2011-12/

[36] ICES. Data, Discovery, Better Health. Accessed August 11, 2021. https://www.ices.on.ca/Data-and-Privacy/ICES-data/Types-of-ICES-Data

[37] Health care in Ontario: OHIP. Accessed August 11, 2021. https://www.ontario.ca/page/health-care-ontario

[38] Ontario Ministry of Health. COVID 19 vaccine guidance. Accessed August 11, 2021. https://www.health.gov.on.ca/en/pro/programs/publichealth/coronavirus/docs/vaccine/COVID-19_vaccine_administration.pdf

[39] Miller A. Canada is flying blind with Omicron as COVID-19 testing drops off a cliff. *CBC*. January 12, 2022. Accessed June 20, 2023. https://www.cbc.ca/news/health/omicron-testing-canada-cases-hospitalizations-po-1.6304195

[40] Moore O, Hager M. Quebec imposes COVID-19 curfew; Ontario limits eligibility for PCR tests amid Omicron surge. *The Globe and Mail*. December 30, 2021. Accessed June 20, 2023. https://www.theglobeandmail.com/canada/article-ontario-quebec-announce-new-measures-to-deal-with-omicron-surge/

[41] Skowronski DM, Kaweski SE, Irvine MA, . Serial cross-sectional estimation of vaccine and infection-induced SARS-CoV-2 sero-prevalence in children and adults, British Columbia, Canada: March 2020 to August 2022. medRxiv. Preprint posted online September 9, 2022. doi:10.1101/2022.09.09.22279751 PMC9828974 36507788

[42] Williams AM, Clayton HB, Singleton JA. Racial and ethnic disparities in COVID-19 vaccination coverage: the contribution of socioeconomic and demographic factors. Am J Prev Med. 2022;62(4):473-482. doi:10.1016/j.amepre.2021.10.008 34872772 PMC8598940

[43] Matheson FI, Dunn JR, Smith KL, Moineddin R, Glazier RH. Development of the Canadian Marginalization Index: a new tool for the study of inequality. Can J Public Health. 2012;103(8)(suppl 2):S12-S16. doi:10.1007/BF03403823 23618065 PMC6973681

[44] ICES. ICES COVID-19 dashboard. Accessed June 20, 2023. https://www.ices.on.ca/DAS/AHRQ/COVID-19-Dashboard

[45] Austin PC. Using the standardized difference to compare the prevalence of a binary variable between two groups in observational research. Commun Stat Simul Comput. 2009;38(6):1228-1234. doi:10.1080/03610910902859574

[46] WHO. Threats to global health. Accessed June 20, 2023. https://www.who.int/news-room/spotlight/ten-threats-to-global-health-in-2019

[47] Betsch C, Schmid P, Heinemeier D, Korn L, Holtmann C, Böhm R. Beyond confidence: development of a measure assessing the 5C psychological antecedents of vaccination. PLoS One. 2018;13(12):e0208601. doi:10.1371/journal.pone.0208601 30532274 PMC6285469

[48] MacDonald NE, Comeau J, Dubé È, . Royal Society of Canada COVID-19 report: enhancing COVID-19 vaccine acceptance in Canada. Facets. 2021;6:1184-1246. doi:10.1139/facets-2021-0037

[49] Ekezie W, Awwad S, Krauchenberg A, ; For The ImmuHubs Consortium. Access to vaccination among disadvantaged, isolated and difficult-to-reach communities in the WHO European region: a systematic review. Vaccines (Basel). 2022;10(7):1038. doi:10.3390/vaccines10071038 35891201 PMC9324407

[50] MacDonald SE, Paudel YR, Du C. COVID-19 vaccine coverage among immigrants and refugees in Alberta: a population-based cross-sectional study. J Glob Health. 2022;12:05053. doi:10.7189/jogh.12.05053 36472928 PMC9725104

[51] Gure Y. Integration outcomes of refugees in Canada: Findings from the 2016 Census. Paper presented at: Pathways to Prosperity Conference; November 8, 2021; online. Accessed June 20, 2023. http://p2pcanada.ca/wp-content/blogs.dir/1/files/2022/01/Yasmin-Gure.pdf

[52] Temsah MH, Alhuzaimi AN, Aljamaan F, . Parental attitudes and hesitancy about COVID-19 vs. routine childhood vaccinations: a national survey. Front Public Health. 2021;9:752323. doi:10.3389/fpubh.2021.752323 34722451 PMC8548678

[53] Xu Y, Xu D, Luo L, . A cross-sectional survey on COVID-19 vaccine hesitancy among parents from Shandong vs. Zhejiang. Front Public Health. 2021;9:779720. doi:10.3389/fpubh.2021.779720 34805084 PMC8602062

[54] Zhou Y, Zhang J, Wu W, Liang M, Wu QS. Willingness to receive future COVID-19 vaccines following the COVID-19 epidemic in Shanghai, China. BMC Public Health. 2021;21(1):1103. doi:10.1186/s12889-021-11174-0 34107930 PMC8188944

[55] Kelly BJ, Southwell BG, McCormack LA, . Predictors of willingness to get a COVID-19 vaccine in the U.S. BMC Infect Dis. 2021;21(1):338. doi:10.1186/s12879-021-06023-9 33845781 PMC8039496

[56] Montalti M, Rallo F, Guaraldi F, . Would parents get their children vaccinated against SARS-CoV-2: rate and predictors of vaccine hesitancy according to a survey over 5000 families from Bologna, Italy. Vaccines (Basel). 2021;9(4):366. doi:10.3390/vaccines9040366 33920109 PMC8069076

[57] Scherer AM, Gedlinske AM, Parker AM, . Acceptability of adolescent COVID-19 vaccination among adolescents and parents of adolescents—United States, April 15-23, 2021. MMWR Morb Mortal Wkly Rep. 2021;70(28):997-1003. doi:10.15585/mmwr.mm7028e1 34264908 PMC8314712

[58] Willis DE, Schootman M, Shah SK, . Parent/guardian intentions to vaccinate children against COVID-19 in the United States. Hum Vaccin Immunother. 2022;18(5):2071078. doi:10.1080/21645515.2022.2071078 35506876 PMC9302502

[59] Debela MS, Garrett APN, Charania NA. Vaccine hesitancy and its determinants among refugee parents resettled in Aotearoa New Zealand. Hum Vaccin Immunother. 2022;18(6):2131336. doi:10.1080/21645515.2022.2131336 36315907 PMC9746517

[60] Our World in Data. Coronavirus (COVID-19) vaccinations. Accessed June 20, 2023. https://ourworldindata.org/covid-vaccinations

[61] Acevedo-Garcia D, Sanchez-Vaznaugh EV, Viruell-Fuentes EA, Almeida J. Integrating social epidemiology into immigrant health research: a cross-national framework. Soc Sci Med. 2012;75(12):2060-2068. doi:10.1016/j.socscimed.2012.04.040 22721965

[62] Nijman RG, Bressan S, Brandenberger J, . Update on the coordinated efforts of looking after the health care needs of children and young people fleeing the conflict zone of Ukraine presenting to European emergency departments—a joint statement of the European Society for Emergency Paediatrics and the European Academy of Paediatrics. Front Pediatr. 2022;10:897803. doi:10.3389/fped.2022.897803 35558376 PMC9090499

[63] Health Cluster Ukraine. Ukraine: Public Health Situation Analysis (PHSA)–short-form. Accessed June 20, 2023. https://www.humanitarianresponse.info/sites/www.humanitarianresponse.info/files/documents/files/ukraine-phsa-shortform-030322.pdf

[64] Wróblewski M, Meler A, Stankowska J, Kawiak-Jawor E. An analysis of factors shaping vaccine attitudes and behaviours in a low-trust society based on structural equation modelling—the case of Poland’s vaccination programme against COVID-19. Int J Environ Res Public Health. 2022;19(22):14655. doi:10.3390/ijerph192214655 36429367 PMC9690255

[65] Milošević Đorđević J, Mari S, Vdović M, Milošević A. Links between conspiracy beliefs, vaccine knowledge, and trust: anti-vaccine behavior of Serbian adults. Soc Sci Med. 2021;277:113930. doi:10.1016/j.socscimed.2021.113930 33873008 PMC8634900

[66] Pollock G, Newbold B, Lafrenière G, Edge S. Perceptions of discrimination in health services expereinced by immigrant minorities in Ontario. Accessed June 20, 2023. http://p2pcanada.ca/files/2015/09/Perceptions-of-Discrimination-in-Health-Services-Experienced-by-Immigrant-Minorities-in-Ontario.pdf

[67] Kemei J, Alaazi DA, Olanlesi-Aliu A, . What contributes to COVID-19 online disinformation among Black Canadians: a qualitative study. CMAJ Open. 2023;11(3):E389-E396. doi:10.9778/cmajo.20220197 37130607 PMC10158753

[68] Ganczak M, Bielecki K, Drozd-Dąbrowska M, . Vaccination concerns, beliefs and practices among Ukrainian migrants in Poland: a qualitative study. BMC Public Health. 2021;21(1):93. doi:10.1186/s12889-020-10105-9 33413287 PMC7789884

[69] Marzo RR, Ahmad A, Islam MS, . Perceived COVID-19 vaccine effectiveness, acceptance, and drivers of vaccination decision-making among the general adult population: a global survey of 20 countries. PLoS Negl Trop Dis. 2022;16(1):e0010103. doi:10.1371/journal.pntd.0010103 35089917 PMC8797205

[70] Abuelezam NN, El-Sayed AM, Galea S. Relevance of the “immigrant health paradox” for the health of Arab Americans in California. Am J Public Health. 2019;109(12):1733-1738. doi:10.2105/AJPH.2019.305308 31622140 PMC6836791

[71] Wang Q, Yang L, Jin H, Lin L. Vaccination against COVID-19: a systematic review and meta-analysis of acceptability and its predictors. Prev Med. 2021;150:106694. doi:10.1016/j.ypmed.2021.106694 34171345 PMC8217737

[72] McKenzie-Sutter H. Ontario regions push to boost youth vaccination rate before students head back to school. *CBC*. July 27, 2021. Accessed June 20, 2023. https://www.cbc.ca/news/canada/toronto/ontario-regions-boost-youth-vaccination-rate-1.6119671

[73] Kholina K, Harmon SHE, Graham JE. An equitable vaccine delivery system: lessons from the COVID-19 vaccine rollout in Canada. PLoS One. 2022;17(12):e0279929. doi:10.1371/journal.pone.0279929 36584230 PMC9803301

[74] Szilagyi PG, Shah MD, Delgado JR, . Parents’ intentions and perceptions about COVID-19 vaccination for their children: results from a national survey. Pediatrics. 2021;148(4):e2021052335. doi:10.1542/peds.2021-052335 34344800 PMC10116994

[75] Musa S, Dergaa I, Abdulmalik MA, Ammar A, Chamari K, Saad HB. BNT162b2 COVID-19 vaccine hesitancy among parents of 4023 young adolescents (12-15 years) in Qatar. Vaccines (Basel). 2021;9(9):981. doi:10.3390/vaccines9090981 34579218 PMC8473301

[76] Dayton L, Miller J, Strickland J, Davey-Rothwell M, Latkin C. A socio-ecological perspective on parents’ intentions to vaccinate their children against COVID-19. Vaccine. 2022;40(32):4432-4439. doi:10.1016/j.vaccine.2022.05.089 35697575 PMC9168003

[77] Altulaihi BA, Alaboodi T, Alharbi KG, Alajmi MS, Alkanhal H, Alshehri A. Perception of parents towards COVID-19 vaccine for children in Saudi population. Cureus. 2021;13(9):e18342. doi:10.7759/cureus.18342 34646710 PMC8481148

[78] A K, Lu X, Wang J, Hu L, Li B, Lu Y. Association between adult vaccine hesitancy and parental acceptance of childhood COVID-19 vaccines: a web-based survey in a northwestern region in China. Vaccines (Basel). 2021;9(10):1088. doi:10.3390/vaccines9101088 34696196 PMC8539638

[79] Goldman RD, Yan TD, Seiler M, ; International COVID-19 Parental Attitude Study (COVIPAS) Group. Caregiver willingness to vaccinate their children against COVID-19: cross sectional survey. Vaccine. 2020;38(48):7668-7673. doi:10.1016/j.vaccine.2020.09.084 33071002 PMC7547568

[80] Khan FL, Nguyen JL, Singh TG, . Estimated BNT162b2 vaccine effectiveness against infection with Delta and Omicron variants among US children 5 to 11 years of age. JAMA Netw Open. 2022;5(12):e2246915. doi:10.1001/jamanetworkopen.2022.46915 36515946 PMC9856252

