# Supplementary material for: COVID-19 Vaccine Uptake in Immigrant, Refugee, and Nonimmigrant Children and Adolescents in Ontario, Canada
Source: JAMA Netw Open. 2023 Jul 26;6(7):e2325636. doi: 10.1001/jamanetworkopen.2023.25636 (PMC10372706; doi:10.1001/jamanetworkopen.2023.25636)
Supplement: Supplement 1. — eTable 1. List of Databases Used in the Study eTable 2. List of Variables Used in the Study eTable 3. Relevant Immigration Pathways in Canada and Context-Specific Definitions eReferences. eTable 4. Additional Baseline Characteristics of Immigrants, Refugees, Second-Generation Immigrants, Refugees, and All Nonimmigrant Children and Adolescents in Ontario on January 1, 2021 eTable 5. Crude and Adjusted Odds Ratios of Being Vaccinated (Full Cohorts) on April 24, 2022 eTable 6. Adjusted Odds Ratios of Being Vaccinated Among First- and Second-Generation Immigrant and Refugee Children on April 24, 2022 eTable 7. Adjusted Odds Ratios of Being Vaccinated Among First- and Second-Generation Immigrant and Refugee Adolescents on April 24, 2022 eFigure 1. Study Population Inclusion Flowchart eFigure 2. Milestones of the Ontario Vaccination Campaign in Relation to the Study Timeline [file jamanetwopen-e2325636-s001.pdf]

Supplemental Online Content

Brandenberger J, Duchen R, Lu H, et al. COVID-19 vaccine uptake in immigrant, refugee, and nonimmigrant children and adolescents in Ontario, Canada. *JAMA Netw Open*. 2023;6(7):e2325636. doi:10.1001/jamanetworkopen.2023.25636

eTable 1. List of Databases Used in the Study
eTable 2. List of Variables Used in the Study
eTable 3. Relevant Immigration Pathways in Canada and Context-Specific Definitions
eReferences.
eTable 4. Additional Baseline Characteristics of Immigrants, Refugees, Second-Generation Immigrants, Refugees, and All Nonimmigrant Children and Adolescents in Ontario on January 1, 2021
eTable 5. Crude and Adjusted Odds Ratios of Being Vaccinated (Full Cohorts) on April 24, 2022
eTable 6. Adjusted Odds Ratios of Being Vaccinated Among First- and Second-Generation Immigrant and Refugee Children on April 24, 2022
eTable 7. Adjusted Odds Ratios of Being Vaccinated Among First- and Second-Generation Immigrant and Refugee Adolescents on April 24, 2022
eFigure 1. Study Population Inclusion Flowchart
eFigure 2. Milestones of the Ontario Vaccination Campaign in Relation to the Study Timeline

This supplemental material has been provided by the authors to give readers additional information about their work.

© 2023 Brandenberger J et al. *JAMA Network Open.*

eTable 1. List of Databases Used in the Study

Name of database	Data provider	Description
Client Agency Program Enrolment (CAPE)	Ontario Ministry of Health	Indicates the enrollment of an individual in a primary care program with a specific practitioner or group.
Community Health Centres (CHCs)	Alliance for Healthier Communities	CHCs deliver primary care services in combination with health promotion and illness prevention services, primarily in high need areas to individuals who do not have a health care provider, are newcomers to Canada, face barriers to care, have mental health or addiction issues, require counseling or help with housing issues, and/or have no health insurance. Care is provided by physicians, nurse practitioners, nurses, counsellors, community workers, and dietitians. The dataset includes chart abstracted records for all visits in all such centres across Ontario. The dataset is compiled and validated by the Alliance for Healthier Communities before being sent to ICES for use in healthcare research. Data are updated annually and records are linked using individuals encrypted health card numbers. None of the care provided by CHCs is captured in OHIP and thus CHC data represents a separate source of primary care access, particularly by vulnerable Ontarians.
Corporate Provider Database (CPDB)	Ontario Ministry of Health	Contains information on providers (e.g., physicians, nurses) and groups (e.g., primary care, hospitals) eligible to receive payment from OHIP.
Discharge Abstract Database (DAD)	Canadian Institute for Health Information (CIHI)	Contains administrative, clinical (diagnoses and procedures/interventions), demographic, and administrative information from all admissions to acute care hospitals in Ontario. CIHI receives records from hospitals, validates and cleans them before being sent to ICES. ICES links consecutive DAD records together to form 'episodes of care' among the hospitals to identify patients who have been transferred after their initial admission. The dataset is updated every quarter, however more timely updates (up to weekly) occurred during the pandemic to facilitate pandemic-related research.
Immigration, Refugees, and Citizenship Canada's Permanent Resident Database	Immigration & Refugees and Citizenship Canada (IRCC)	Permanent residents' demographic information collected by the IRCC during the immigration application process. Overall 86.4% of immigrants and 92% of refugees in the IRCC database were linked to the healthcare registry, with minimal variability by immigration category, region of birth and minimal differences in characteristics between linked and unlinked people[1].
Mother-Baby (MOMBABY)	ICES derived cohort	Links Discharge Abstract Database (DAD) inpatient records of delivering mothers and their newborns. Each record corresponds to a mother-child pair.
National Ambulatory Care Reporting System (NACRS)	Canadian Institute for Health Information (CIHI)	Contains administrative, clinical (diagnoses and procedures), demographic, and administrative information for all visits made to hospital- and community-based ambulatory care centres (emergency departments, day surgery units, hemodialysis units, and cancer care clinics) . CIHI receives records from hospitals, validates and cleans them before being sent to ICES. The dataset

© 2023 Brandenberger J et al. *JAMA Network Open.*

Name of database	Data provider	Description
		is updated every quarter, however more timely updates (up to weekly) occurred during the pandemic to facilitate pandemic-related research.
Ontario Health Insurance Program (OHIP) Registered Persons Database (RPDB) (healthcare registry)	Ontario Ministry of Health	Basic demographic information (age, sex, location of residence, date of birth, and date of death for deceased individuals) for all individuals issued an Ontario health insurance number. Also indicates the time periods individuals are eligible for health insurance and provides the best-known postal code for each registrant on July 1st of each year which is updated yearly. Linkable to all other ICES data holdings using an encrypted health card number.
Ontario Asthma Datbaset (ASTHMA)	ICES derived cohort	Individuals were classified as having asthma based on an algorithm of 2 or more physician billings (OHIP) within 2 years or one hospitalization (DAD) with an asthma diagnosis code prior to index date[2].
Ontario COVID-19 vaccine data (COVaxON)	Ontario Ministry of Health<br><br>Public Health Ontario (PHO)	The central provincial database and point-of-care system supporting COVID-19 vaccination. The Ministry of Health compiles and manages these individual level data and Public Health Ontario provide ICES with a client-level dataset detailing vaccination records. While vaccination records have been cleaned using data cleaning logic, in instances where multiple records remain present for an individual, the records are reconciled manually based on the most likely scenario (1. 2nd record represents a second vaccination event [i.e. 2nd dose]; 2. Repeat record; 3. Incorrectly entered identifier (in which case the earliest record was selected)
Ontario Crohn's and Colitis Cohort Database (OCCC)	ICES derived cohort	Individuals were diagnosed with inflammatory bowel disease (IBD) if they had: one procedure code for sigmoidoscopy/colonoscopy (OHIP); or 2 hospitalizations (DAD) or 4 physician billings (OHPI)/emergency department visits (NACRS) with IDB diagnosis code within 3 years (based on at least 2 years of OHIP eligibility[3, 4].
Ontario Diabetes Database (ODD)	ICES derived cohort	Individuals were diagnosed with diabetes based on 1 or more physician billings with a diabetes fee code prior to index date[5].
Ontario Health Insurance Plan (OHIP)	Ontario Ministry of Health	Contains information on inpatient and outpatient services provided to Ontario residents eligible for health insurance by fee-for-service health care practitioners (primarily physicians) and "shadow billings" for those paid through non-fee-for-service payment plans. Billing codes on the claims (OHIP fee codes) identify the care provider, their area of specialization and the type and location of service. OHIP billing claims also contain a 3-digit diagnosis code – the main reason for the service – captured using a modified version of the ICD, 8th revision coding system.
Ontario HIV Database	ICES derived cohort	HIV diagnosis 3 physician billings (OHIP) with HIV diagnosis within 3 years[6].
Ontario Laboratories Information System (OLIS)	Ministry of Health<br><br>eHealth Ontario	Contains lab orders, test requests and lab results from most laboratories in Ontario. Starting April 7 2020, ICES began receiving daily cumulative updates of COVID-19 test orders from eHealth Ontario (eHO, now part of Ontario Health Digital Health Services). These data are a minimum dataset extracted from lab orders with COVID-19-specific test request (TR) or Logical Observation Identifiers Names and Codes (LOINC) and other TR/LOINC codes indicative of viral or respiratory virus testing. Each record represents a testing event, with testing events linkable by encrypted individual health card numbers.

© 2023 Brandenberger J et al. *JAMA Network Open.*

Name of database	Data provider	Description
Ontario Marginalization Index (ON-MARG)	Toronto Community Health Profiles	A geographically (census) based index developed to quantity the degree of marginalization occurring across the province of Ontario. It is comprised of 4 major dimensions though to underlie the construct of marginalization: residential instability, material deprivation, dependency and ethnic concentration. Based on residential postal code as recorded in RPDB[7].
Postal Code Conversion File (PCCF)+	Statistics Canada	A conversion template between the six-character postal code and Statistics Canada's standard geographic areas. Through the link between postal codes and standard geographic areas, the PCCF permits the integration of data from various census derived.
Public Health Case and Contact Management (CCM) Solutions	Public Health Ontario (PHO)	A central data repository for COVID-19 case and contact management, and reporting in Ontario. The Health Protection and Promotion Act requires that each public health unit in Ontario collect information about people with diseases of public health significance (reportable diseases), including COVID-19, in their jurisdiction and report it to the Ministry of Health (MOH). This information is used for local, provincial and national surveillance.
Same Day Surgery	Canadian Institute for Health Information (CIHI)	Contains chart abstract information for all same-day surgeries or procedures performed at acute care hospitals. CIHI receives records from hospitals, validates and cleans them before being sent to ICES. The dataset is updated every quarter, however more timely updates (up to weekly) occurred during the pandemic to facilitate pandemic-related research.

© 2023 Brandenberger J et al. *JAMA Network Open.*

eTable 2. List of Variables Used in the Study

Variables used in this study, data extraction date	Database source	Variable definition and categories	Time of data entry/latest update
Age as of Jan 1st, 2021	OHIP Registered Persons Database (RPDB) (healthcare registry)	Age of the individual at index, calculated from date of birth recorded on the individuals' health card. Categories for this variable include 4-7 years and 8-10 years for children and 11-14 years and 15-17 years for adolescents	Health care registration/renewal
Sex as of Jan 1st, 2021	OHIP Registered Persons Database (RPDB) (healthcare registry)	Biological sex as recorded in the RPDP database.	Health care registration/renewal
Non-immigrants as of Jan 1st, 2021	OHIP Registered Persons Database (RPDB) (healthcare registry)	This variable includes individuals who were born in Ontario, immigrants and refugees who landed in Ontario before Jan 1, 1985 and individuals who landed in Canada via another province and subsequently moved to Ontario	Health care registration/renewal
Immigration category as of Jan 1st, 2021	Immigration, Refugees, and Citizenship Canada's (IRCC) Permanent Resident Database	Immigration category as recorded in the immigration application of the minor (=first generation immigrant) or as recorded in the immigration application of the mother (=second generation immigrant; see below and in eTable2 Supplement). Categories include:<br>1) immigrants (sponsored family immigrants and economic immigrants)<br>2) resettled refugees (privately sponsored refugees, blended visa office-referred refugees, government-assisted refugees)<br>3) protected persons/refugees and other (successful asylum seekers, their dependents, humanitarian and compassionate/public policy cases)[17]	Time of immigration application of minor/mother, last updated in September 2020
Second generation immigrant/refugee	Mother-Baby (MOMBABY)	Minors born in Ontario to women and other birthing people who immigrated on or after Jan 1st, 1985	Admission for childbirth
Region of origin	Immigration, Refugees, and Citizenship Canada's (IRCC) Permanent Resident Database	Country of origin of an immigrant/refugee (first generation) or of the mother (second generation immigrant/refugee), further aggregated into world regions using the World Bank classification system[8]. Categories include on the Eastern hemisphere: North Africa, East Africa, Western Africa, Central Africa, Southern Africa, Middle East, East Asia, South Asia, Southeast Asia and Australasia & Oceania and on the Western hemisphere: North America, Central America, Caribbean, South America, Eastern Europe and Europe, other. The small number of subjects for which region of origin was not stated/missing (n=9) were excluded from the final analysis	Time of immigration application
Recency of immigration as of January 1st, 2021	Immigration, Refugees, and Citizenship Canada's (IRCC) Permanent Resident Database	Time interval since immigration as difference between index date and earliest of either first OHIP eligibility or arrival date. Categories include recent (0 to ≤ 5 years), intermediate (> 5 years and ≤10 years) long-term (> 10 years)	Arrival date/OHIP registration
Rural Residence as of Jan first, 2021	Statistics Canada's Postal Code Conversion File (PCCF)+<br><br>OHIP Registered Persons Database (RPDB)	Location of residence defined by the most recent postal code, further classified as binary variable (rural o/urban) with rural being defined as communities of < 10,000 persons<br>The small number of subjects (children: n=1,980 (0.2%); adolescents: n=1,463 (0.2%)) with missing data were excluded from the final models.	2016 Census Canada

© 2023 Brandenberger J et al. *JAMA Network Open.*

Variables used in this study, data extraction date	Database source	Variable definition and categories	Time of data entry/latest update
	(healthcare registry)		
Primary care access model as of Jan 1st , 2021	Client Agency Program Enrolment (CAPE)<br><br>Corporate Provider Database (CPDB)<br><br>CHC<br><br>Ontario Health Insurance Plan (OHIP)	The primary care access model describes the primary care model recorded for the individual based on rostering and primary care use one year prior to January 1st 2021<br>As done similarly in previous studies[9], the primary care access model was assigned in a stepwise approach. First all individuals who used a community health centre (CHC) were identified and assigned to the category "CHC". In a second step, the rostering data from CAPE was used to describe those enrolled with family physicians who are part of patient enrollment models. In a third step, the remaining individuals were virtually assigned to the primary care provider (includes family physicians and pediatricians) who billed the highest amount of money for primary care visits one year prior to January 1st 2021. If there were no primary care visits, the individual was assigned to the category: "no regular provider". Categories included:<br><br>*Community Health Centre*<br><br>*Rostered primary care model*<br><br>*Pediatrician*<br><br>*Non-comprehensive care*: fee-for-service practices of general practitioner/family physicians who are usually solo-practitioners, or those whose practices do not provide comprehensive services, or walk-in clinics, and does not belong to an enrolment group<br><br>*No regular provider*: includes non-rostered individuals and others with no primary care visits in the previous year<br>The CAPE data are updated bimonthly.	Jan 1, 2021
Influenza vaccination in 2019/2020	Ontario Health Insurance Plan (OHIP)	Physician, nurse, or pharmacist billings for influenza vaccination in Winter 2019/2020	Winter 2019/2020
Pediatric chronic condition[10] as of Jan 1st , 2021	Discharge Abstract Database (DAD)<br><br>National Ambulatory Care Reporting System (NACRS)<br><br>ODD<br><br>ASTHMA<br><br>OCCC<br><br>OHIP<br><br>Same Day Surgery	Chronic conditions were assigned based on presence in the available validated chronic disease registry (diabetes, asthma, inflammatory bowel disease, HIV). The presence of a pediatric complex chronic condition (neurological/neuromuscular, cardiovascular, respiratory, uro-renal & gastrointestinal, hematological and immunodeficiency, metabolic, premature/neonatal) was assigned using health records back to birth or the earliest records in Ontario using predefined, standardized definitions[11]. All conditions were aggregated into one binary variable (categories yes/no).	Jan 1st, 2021

© 2023 Brandenberger J et al. *JAMA Network Open.*

Variables used in this study, data extraction date	Database source	Variable definition and categories	Time of data entry/latest update
COVID-19 vaccination as of April 24th 2022	Ontario COVID-19 vaccine data (COVaxON)	Vaccination was defined for children (4-10 on index date) as at least one dose of any COVID 19 vaccination and for adolescents (11-17 on index date) as at least:<br>- two doses of COVID-19 vaccines authorized by Health Canada (e.g., those produced by Pfizer-BioNTech or Moderna), OR<br>- one dose of a non-Health Canada authorized COVID-19 vaccine (e.g. CoronaVac/Sinovac and Sinopharm/BBIBP, or Sputnik V) AND one dose of a Health Canada authorized COVID-19 vaccine, OR<br>- three doses of any COVID-19 vaccine (whether or not the vaccines are authorized by Health Canada)<br><br>Children aged 5-11 became eligible on November, 25th 2021 for the children's vaccination. Adolescents aged 12-17 became eligible on May 23rd 2021 for the adolescent vaccination. In autumn 2021, 11 year olds became eligible for the adolescent vaccination before their 12th birthday as long as they turned 12 in 2021.	Adolescents: May 23rd[12] - April 24th 2022<br><br>Children: November 25th[13] 2021 - April 24th 2022
Neighbourhood COVID-19 risk decile	Ontario Laboratories Information System (OLIS)<br><br>Public Health Case and Contact Management (CCM) Solutions	Cumulative incidence of COVID-19 cases for each Forward Sortation Area (FSA; consisting of an average of 8000 households) from individuals living outside of long-term care facilities. FSAs were ranked by their cumulative incidence and grouped into deciles so that each decile had 10% of the population. Each decile represents the neighbourhood risk of COVID-19, where 1 = highest incidence neighbourhoods and 10 = lowest incidence neighbourhoods.<br>The small number of individuals with missing data (children: n=32 (0.003%); adolescents: n=31 (0.003%)) were excluded from the final models	March 28, 2021
Previous COVID-19 infection<br><br>as of May 23rd, 2021 for adolescents and of November 25th, 2021 for children	Ontario Laboratories Information System (OLIS)<br><br>Distributed testing data from laboratories (DL)<br><br>Public Health Case and Contact Management (CCM) Solutions	Previous COVID19 infections were based on a comprehensive database that included information on PCR COVID 19 tests (OLIS and DL) and public health contact tracing information (CCM)<br>Infections were assessed prior to May, 23rd 2021 for adolescents and prior to November 25th 2021.<br>OLIS/DL is updated on a monthly base, CCM is updated weekly.	Adolescents: prior to May 23rd, 2021<br><br>Children: prior to November 25th, 2021
Material Deprivation Quintile as of Jan 1st , 2021	Ontario Marginalization Index	Material Deprivation is a construct from the Ontario Marginalization Index[14] and includes the proportion of:<br>- Lone-parent families<br>- Population aged 25+ without post secondary education<br>- Population receiving govenrment transfer payments<br>- Population aged 15+ who are unemployed<br>- Low-income population<br>- Dwellings in need of major repair[15]	2016 Census Canada

© 2023 Brandenberger J et al. *JAMA Network Open.*

Variables used in this study, data extraction date	Database source	Variable definition and categories	Time of data entry/latest update
		It is categorized from Q1 [lowest deprivation] to Q5 [highest deprivation]. Includes calculated from the Canada Census at the level of a dissemination area (400-700 inhabitants). Neighbourhoods with unstable populations are suppressed and known to be highly deprived. Therefore, the small numbers of subjects with suppressed material deprivation were merged with the most deprived quintile. The number of individuals with suppressed data were: children: n=12,915 (1.2%) adolescents: n=12,541 (1.1%).	

© 2023 Brandenberger J et al. *JAMA Network Open.*

eTable 3. Relevant Immigration Pathways in Canada and Context-Specific Definitions

**First Generation Immigrants/ Refugees**	Minors (<18 years) born outside of the country who crossed an international border to live in Canada. Immigrants: minors accepted as permanent residents, refugee: minors accepted permanent residents fulfilling the UNHCR definition[b]	**Second-Generation Immigrants/ Refugees**	Minors born in Ontario to mothers/birthing parents who were immigrants or refugees who arrived in Ontario since 1985	**Non-Immigrant Children and Adolescents**	Minors living in Ontario who were not identified as first or second generation immigrants/refugees

**Immigrants (83.1% of immigrants/refugees)[a]**		**Resettled Refugees[b] (7.4% of immigrants/refugees)[a]**		
**Sponsored family**	**Economic class**	**Privately sponsored refugee**	**Blended visa office-referred refugees**	**Government-assisted refugees**
Sponsored by a family member who is a Canadian citizen or permanent resident of Canada and aged 18 years or older. Around 14% of sponsored family members are children (0-9years) and 15% adolescents (10-19 years)[a]	Selected based on skills and ability to contribute to Canada's economy. Includes skilled workers, business immigrants, provincial or territorial nominees and those in the Canadian Experience Class (a permanent residence category for people with one year of skilled work experience in Canada). Around 9 % are children(0-9 years) and 12% adolescents (10-19 years)[a]	Privately sponsored by organizations, individuals or groups of individuals in Canada. Receive resettlement assistance and income support from the sponsoring individuals or groups in the first year. Around 10% are children (0-9 years) and 11% adolescents (10-19 years)[a]	Sponsored by a partnership of the Government of Canada, the United Nations High Commissioner for Refugees and private organizations. Receive income support from the federal government in first 6 months and resettlement assistance and income support from private sponsors in the first year. The program started in 2013. Around 29% are children (0-9 years) and 27% adolescents (10-19 years)[a]	Sponsored by and receive resettlement assistance and income support from the Government of Canada in the first year. Around 14% are children (0-9 years) and 15% adolescents (10-19 years)[a]

**Protected Persons (6.0% of immigrants/ refugees)[a]**	**Others (3.5% of immigrants/refugees)[a]**		
**Refugees* landed in Canada (asylum seekers or refugee claimants)**	**Refugee* dependents**	**Humanitarian and compassionate or public policy cases**	**Other immigrants**
Individuals who applied for refugee protection while in Canada and were granted permanent resident status on the basis of a well-founded fear of returning to their country of origin. Not sponsored by the federal government or a private group. Around 10% are children (0-9 years) and 12% adolescents (10-19 years)[a]	Family members of a protected person in Canada, who were living abroad at the time of application and whose application for permanent residence was considered concurrently with that of the protected person in Canada. Around 15% are children (0-9 years) and 17% adolescents (10-19 years)[a]	Sponsored and unsponsored individuals who would not otherwise qualify in any category, in cases where there are strong humanitarian and compassionate considerations, or for public policy reasons. Around 10% are children (0-9 years) and 10% adolescents (10-19 years)[a] .	Post-determination refugee claimants in Canada, deferred removal orders and retirees. Around 3% are children (0-9 years) and 7% adolescents (10-19 years)[a]

[a]Proportions are from an ICES report which describes first generation refugees and immigrants who landed in Ontario between 1st of January 1985 and 31st of May 2017 as well as second generation immigrant children and adolescents as of March 31, 2020 where their maternal/birthing parent's immigration category has been assigned.[16]
[b]The United Nations High Commissioner for Refugees (UNHCR) defines a refugee as "someone who is unable or unwilling to return to their country of origin owing to a well-founded fear of being persecuted for reasons of race, religion, nationality, membership of a particular social group, or political opinion

© 2023 Brandenberger J et al. *JAMA Network Open.*

eReferences

1. Chiu M, Lebenbaum M, Lam K, et al. Describing the linkages of the immigration, refugees and citizenship Canada permanent resident data and vital statistics death registry to Ontario's administrative health database. *BMC Medical Informatics and Decision Making*. 2016/10/21 2016;16(1):135. doi:10.1186/s12911-016-0375-3
2. Gershon AS, Wang C, Guan J, Vasilevska-Ristovska J, Cicutto L, To T. Identifying patients with physician-diagnosed asthma in health administrative databases. *Can Respir J*. Nov-Dec 2009;16(6):183-8. doi:10.1155/2009/963098
3. Benchimol EI, Guttmann A, Griffiths AM, et al. Increasing incidence of paediatric inflammatory bowel disease in Ontario, Canada: evidence from health administrative data. *Gut*. Nov 2009;58(11):1490-7. doi:10.1136/gut.2009.188383
4. Benchimol EI, Guttmann A, Mack DR, et al. Validation of international algorithms to identify adults with inflammatory bowel disease in health administrative data from Ontario, Canada. *J Clin Epidemiol*. Aug 2014;67(8):887-96. doi:10.1016/j.jclinepi.2014.02.019
5. Hux JE, Ivis F, Flintoft V, Bica A. Diabetes in Ontario: determination of prevalence and incidence using a validated administrative data algorithm. *Diabetes Care*. Mar 2002;25(3):512-6. doi:10.2337/diacare.25.3.512
6. Antoniou T, Zagorski B, Loutfy MR, Strike C, Glazier RH. Validation of case-finding algorithms derived from administrative data for identifying adults living with human immunodeficiency virus infection. *PLoS One*. 2011;6(6):e21748. doi:10.1371/journal.pone.0021748
7. Matheson FI, Dunn JR, Smith KL, Moineddin R, Glazier RH. Development of the Canadian Marginalization Index: a new tool for the study of inequality. *Can J Public Health*. Apr 30 2012;103(8 Suppl 2):S12-6. doi:10.1007/bf03403823
8. Standard country or area codes for statistical use (M49). https://unstats.un.org/unsd/methodology/m49/.
9. Gill PJ, Saunders N, Gandhi S, et al. Emergency Department as a First Contact for Mental Health Problems in Children and Youth. *J Am Acad Child Adolesc Psychiatry*. Jun 2017;56(6):475-482.e4. doi:10.1016/j.jaac.2017.03.012
10. Feudtner C, Feinstein JA, Zhong W, Hall M, Dai D. Pediatric complex chronic conditions classification system version 2: updated for ICD-10 and complex medical technology dependence and transplantation. *BMC Pediatr*. Aug 8 2014;14:199. doi:10.1186/1471-2431-14-199
11. ICES. ICES COVID-19 Dashboard, Applied Health Research Questions (AHRQ) # 2021 0950 080 000, Definitions, line 136-192. Accessed 13.12.2022. https://urldefense.com/v3/__https:/www.ices.on.ca/*/media/Files/COVID-19/ICES-COVID19-Vaccine-Coverage-by-Priority-Group.ashx?la=en-CA__;fg!!D0zGoin7BXfl!4j8tM7CVJU7-z3tLxGzNI533JXWHhoeXctsoPUQdp-vLf-Sz4hFT_ILKje-AM3Vkv9oLfgK4_cNYiNI9p9QzQqwlkksJIYD1Nsd7$
12. COVID-19 Vaccine Booking Expanding to Youth 12+ Ahead of Schedule. https://news.ontario.ca/en/release/1000185/covid-19-vaccine-booking-expanding-to-youth-12-ahead-of-schedule. Ontario Newsroom.
13. Health Canada authorizes use of Comirnaty (the Pfizer-BioNTech COVID-19 vaccine) in children 5 to 11 years of age. https://www.canada.ca/en/health-canada/news/2021/11/health-canada-authorizes-use-of-comirnaty-the-pfizer-biontech-covid-19-vaccine-in-children-5-to-11-years-of-age.html.
14. Ontario Marginalization Index (ON-Marg). https://www.publichealthontario.ca/en/data-and-analysis/health-equity/ontario-marginalization-index.
15. Canadian/Ontario Marginalization Index. http://stmichaelshospitalresearch.ca/research-programs/urban-health-solutions/resources-and-reports/canadianontario-marginalization-index/
16. ICES. *COVID 19 in Immigrants, Refugees and Other Newcomers in Ontario: Characteristics of Those Tested and Those Confirmed Positive, as of June 13, 2020* 2020. https://www.ices.on.ca/Publications/Atlases-and-Reports/2020/COVID-19-in-Immigrants-Refugees-and-Other-Newcomers-in-Ontario
17. Statistics Canada. 2016 Census of population. Accessed March 2, 2023. https://www12.statcan.gc.ca/census-recensement/2016/index-eng.cfm

© 2023 Brandenberger J et al. *JAMA Network Open.*

eTable 4. Additional Baseline Characteristics of Immigrants, Refugees, Second-Generation Immigrants, Refugees, and All Nonimmigrant Children and Adolescents in Ontario on January 1, 2021

N (Column %) unless otherwise indicated

Children													
	First generation immigrants/refugees					Second generation immigrants/refugees[e]						Non-immigrants	
	Immigrants[b]	Refugees[c]	SD[a]	Protected persons[d]	SD	Immigrants	SD	Refugees	SD	Protected persons	SD	Non-immigrants	SD
**Rural residence**													
**No**	36,007 (98.4)	10,003 (98.6)	0.015	6,302 (99.1)	0.057	214,126 (98.2)	0.019	14,571 (98.5)	0.002	23,684 (98.7)	0.024	679,124 (86.1)	0.474
**Yes**	536 (1.5)	105 (1.0)	0.039	47 (0.7)	0.07	3,679 (1.7)	0.018	207 (1.4)	0.006	258 (1.1)	0.035	108,120 (13.7)	0.475
**Missing**	41 (0.1)	36 (0.4)	0.05	13 (0.2)	0.023	288 (0.1)	0.006	22 (0.1)	0.01	51 (0.2)	0.025	1,529 (0.2)	0.021
**COVID-19 Neighbourhood Risk Decile**													
**1 (most at risk)**	7,333 (20.0)	955 (9.4)	0.303	1,328 (20.9)	0.021	50,136 (23.0)	0.072	2,449 (16.5)	0.091	5,973 (24.9)	0.116	54,375 (6.9)	0.393
**2**	5,853 (16.0)	1,337 (13.2)	0.08	1,290 (20.3)	0.111	39,284 (18.0)	0.054	1,892 (12.8)	0.092	4,794 (20.0)	0.104	55,467 (7.0)	0.284
**3**	5,407 (14.8)	1,983 (19.5)	0.127	1,047 (16.5)	0.046	29,841 (13.7)	0.031	2,498 (16.9)	0.058	3,383 (14.1)	0.019	65,446 (8.3)	0.204
**4**	4,108 (11.2)	1,913 (18.9)	0.215	834 (13.1)	0.058	21,152 (9.7)	0.05	2,204 (14.9)	0.109	2,495 (10.4)	0.027	75,763 (9.6)	0.053
**5**	2,837 (7.8)	1,043 (10.3)	0.088	416 (6.5)	0.047	19,881 (9.1)	0.049	1,713 (11.6)	0.13	2,282 (9.5)	0.063	83,092 (10.5)	0.097
**6**	3,042 (8.3)	864 (8.5)	0.007	503 (7.9)	0.015	17,657 (8.1)	0.008	1,244 (8.4)	0.003	1,810 (7.5)	0.029	85,052 (10.8)	0.084
**7**	3,770 (10.3)	616 (6.1)	0.155	410 (6.4)	0.14	16,080 (7.4)	0.103	956 (6.5)	0.139	1,324 (5.5)	0.178	79,319 (10.1)	0.008
**8**	2,697 (7.4)	790 (7.8)	0.016	341 (5.4)	0.082	14,711 (6.7)	0.024	1,164 (7.9)	0.019	1,218 (5.1)	0.095	90,587 (11.5)	0.141
**9**	981 (2.7)	348 (3.4)	0.044	151 (2.4)	0.02	5,848 (2.7)	0	508 (3.4)	0.044	536 (2.2)	0.029	102,255 (13.0)	0.39
**10 (least at risk)**	556 (1.5)	295 (2.9)	0.094	42 (0.7)	0.083	3,495 (1.6)	0.007	172 (1.2)	0.031	177 (0.7)	0.074	97,394 (12.3)	0.436
**Missing**	0	0	N/A	0	N/A	8	0.009	0	N/A	1-5	0.009	23	0.008

© 2023 Brandenberger J et al. *JAMA Network Open.*

Children													
	First generation immigrants/refugees					Second generation immigrants/refugees[e]						Non-immigrants	
	Immigrants[b]	Refugees[c]	SD[a]	Protected persons[d]	SD	Immigrants	SD	Refugees	SD	Protected persons	SD	Non-immigrants	SD
**Had a previous COVID-19 infection**	1,191 (3.3)	681 (6.7)	0.159	387 (6.1)	0.134	8,278 (3.8)	0.029	764 (5.2)	0.095	1,109 (4.6)	0.07	19,547 (2.5)	0.047
**Has a pediatric chronic condition**	3,163 (8.6)	881 (8.7)	0.001	497 (7.8)	0.03	52,349 (24.0)	0.425	3,233 (21.8)	0.374	6,306 (26.3)	0.478	140,968 (17.9)	0.275
**Primary care access model**													
**Community Health Centre**	656 (1.8)	1,148 (11.3)	0.392	588 (9.2)	0.331	2,646 (1.2)	0.048	570 (3.9)	0.125	878 (3.7)	0.115	17,151 (2.2)	0.027
**Rostered to a primary care provider**	24,448 (66.8)	6,047 (59.6)	0.15	3,771 (59.3)	0.157	133,409 (61.2)	0.118	8,574 (57.9)	0.184	13,566 (56.5)	0.213	534,379 (67.7)	0.02
**Pediatrican**	1,233 (3.4)	390 (3.8)	0.025	161 (2.5)	0.05	21,403 (9.8)	0.262	1,262 (8.5)	0.219	2,085 (8.7)	0.225	52,161 (6.6)	0.149
**Non-comprehensive care**	6,808 (18.6)	2,049 (20.2)	0.04	1,390 (21.8)	0.081	39,830 (18.3)	0.009	3,027 (20.5)	0.047	5,004 (20.9)	0.056	105,120 (13.3)	0.145
**No regular care provider**	3,439 (9.4)	510 (5.0)	0.17	452 (7.1)	0.083	20,805 (9.5)	0.005	1,367 (9.2)	0.006	2,460 (10.3)	0.029	79,962 (10.1)	0.025
**Influenza vaccination in 2019/2020**	5,571 (15.2)	1,630 (16.1)	0.023	663 (10.4)	0.144	27,916 (12.8)	0.07	1,335 (9.0)	0.191	2,192 (9.1)	0.187	88,277 (11.2)	0.119
**Recency of immigration**													
**Recent (0 to <=5 years)**	20,836 (57.0)	8,568 (84.5)	0.634	4,958 (77.9)	0.459	N/A	N/A	N/A	N/A	N/A	N/A	N/A	N/A
**Intermediate (>5 to <=10 years)**	15,445 (42.2)	1,558 (15.4)	0.621	1,398 (22.0)	0.444	N/A	N/A	N/A	N/A	N/A	N/A	N/A	N/A
**Long-term (>10 years)**	303 (0.8)	18 (0.2)	0.092	6 (0.1)	0.108	N/A	N/A	N/A	N/A	N/A	N/A	N/A	N/A

[a]Standardized differences in comparison with immigrants
[b]Immigrants include economic immigrants and sponsored family immigrants,
[c]Resettled Refugees, include privately sponsored and government assisted refugees,
[d]Protected Persons and Others includes successful asylum seekers and their dependents
[e]Immigrant -related characteristics of second generation immigrants/refugees are those of their mother/birthing parent
Small cells (<6) suppressed, and in the case of non-missing data other cells may be reported as ranges without percentage to reduce risk of re-identification in accordance with ICES policy
N/A= not applicable

© 2023 Brandenberger J et al. *JAMA Network Open.*

Adolescents													
	First generation immigrants/refugees					Second generation immigrants/refugees[e]						Non-immigrants	
	Immigrants[b]	Refugees[c]	SD[a]	Protected persons[d]	SD	Immigrants	SD	Refugees	SD	Protected persons	SD	Non-immigrants	SD
**Rural residence**													
**No**	74,611 (98.6)	13,936 (98.9)	0.03	15,076 (99.0)	0.038	189,196 (98.3)	0.027	11,355 (98.4)	0.019	17,748 (99.0)	0.036	707,011 (86.7)	0.47
**Yes**	897 (1.2)	92 (0.7)	0.056	103 (0.7)	0.053	3,025 (1.6)	0.033	170 (1.5)	0.025	152 (0.8)	0.034	106,695 (13.1)	0.48
**Missing**	157 (0.2)	57 (0.4)	0.036	46 (0.3)	0.019	291 (0.2)	0.013	17 (0.1)	0.014	27 (0.2)	0.013	1,767 (0.2)	0
**COVID-19 Neighbourhood Risk Decile**													
**1 (most at risk)**	15,476 (20.5)	1,769 (12.6)	0.214	3,451 (22.7)	0.054	47,119 (24.5)	0.096	1,994 (17.3)	0.081	4,571 (25.5)	0.12	55,512 (6.8)	0.406
**2**	13,099 (17.3)	1,831 (13.0)	0.12	2,908 (19.1)	0.046	36,353 (18.9)	0.041	1,652 (14.3)	0.082	3,709 (20.7)	0.086	59,261 (7.3)	0.31
**3**	11,370 (15.0)	2,641 (18.8)	0.1	2,420 (15.9)	0.024	26,140 (13.6)	0.041	1,786 (15.5)	0.012	2,662 (14.8)	0.005	67,483 (8.3)	0.212
**4**	8,542 (11.3)	2,650 (18.8)	0.212	1,836 (12.1)	0.024	18,586 (9.7)	0.053	1,562 (13.5)	0.068	1,762 (9.8)	0.048	77,143 (9.5)	0.06
**5**	5,797 (7.7)	1,486 (10.6)	0.101	1,168 (7.7)	0.00	16,710 (8.7)	0.037	1,267 (11.0)	0.114	1,593 (8.9)	0.044	85,806 (10.5)	0.1
**6**	6,171 (8.2)	1,242 (8.8)	0.024	1,221 (8.0)	0.005	14,712 (7.6)	0.019	1,082 (9.4)	0.043	1,318 (7.4)	0.03	89,461 (11.0)	0.096
**7**	6,680 (8.8)	760 (5.4)	0.134	916 (6.0)	0.107	13,168 (6.8)	0.074	743 (6.4)	0.09	918 (5.1)	0.146	83,459 (10.2)	0.048
**8**	5,458 (7.2)	982 (7.0)	0.009	830 (5.5)	0.072	12,013 (6.2)	0.039	918 (8.0)	0.028	910 (5.1)	0.089	96,106 (11.8)	0.156
**9**	1,893 (2.5)	405 (2.9)	0.023	359 (2.4)	0.009	4,815 (2.5)	0.00	414 (3.6)	0.063	354 (2.0)	0.036	104,506 (12.8)	0.395
**10 (least at risk)**	1,176 (1.6)	319 (2.3)	0.052	116 (0.8)	0.074	2,891 (1.5)	0.004	124 (1.1)	0.042	130 (0.7)	0.078	96,713 (11.9)	0.421
**Missing**	1-5	0 (0.0)	0.009	0 (0.0)	0.009	1-5	0.002	0 (0.0)	0.009	0 (0.0)	0.009	23 (0.0)	0
**Had a previous COVID-19 Infection**	2,707 (3.6)	1,085 (7.7)	0.18	939 (6.2)	0.121	7,915 (4.1)	0.028	634 (5.5)	0.092	934 (5.2)	0.08	17,235 (2.1)	0.09

© 2023 Brandenberger J et al. *JAMA Network Open.*

Adolescents													
	First generation immigrants/refugees					Second generation immigrants/refugees[e]						Non-immigrants	
	Immigrants[b]	Refugees[c]	SD[a]	Protected persons[d]	SD	Immigrants	SD	Refugees	SD	Protected persons	SD	Non-immigrants	SD
**Has a pediatric chronic condition**	7,762 (10.3)	1,042 (7.4)	0.101	1,405 (9.2)	0.035	56,427 (29.3)	0.493	3,160 (27.4)	0.449	5,968 (33.3)	0.581	193,997 (23.8)	0.366
**Primary care access model**													
**Community Health Centre**	1,014 (1.3)	1,521 (10.8)	0.404	1,121 (7.4)	0.298	2,054 (1.1)	0.025	340 (2.9)	0.111	460 (2.6)	0.089	16,845 (2.1)	0.056
**Rostered to a primary care provider**	53,434 (70.6)	8,613 (61.2)	0.201	9,456 (62.1)	0.181	137,302 (71.3)	0.015	7,699 (66.7)	0.084	11,963 (66.7)	0.084	603,001 (73.9)	0.074
**Pediatrican**	1,162 (1.5)	277 (2.0)	0.033	236 (1.6)	0.001	9,428 (4.9)	0.191	552 (4.8)	0.186	871 (4.9)	0.19	32,726 (4.0)	0.151
**Non-comprehensive care**	10,183 (13.5)	2,641 (18.8)	0.144	2,897 (19.0)	0.151	24,250 (12.6)	0.026	1,751 (15.2)	0.049	2,623 (14.6)	0.034	78,266 (9.6)	0.121
**No regular care provider**	9,872 (13.0)	1,033 (7.3)	0.19	1,515 (10.0)	0.097	19,478 (10.1)	0.092	1,200 (10.4)	0.082	2,010 (11.2)	0.056	84,635 (10.4)	0.083
**Influenza vaccination in 2019/2020**	5,216 (6.9)	1,527 (10.8)	0.139	1,030 (6.8)	0.005	12,990 (6.7)	0.006	581 (5.0)	0.079	903 (5.0)	0.078	37,253 (4.6)	0.1
**Recency of immigration**													
**Recent (0 to <=5 years)**	14,393 (19.0)	8,474 (60.2)	0.927	6,165 (40.5)	0.483	N/A	N/A	N/A	N/A	N/A	N/A	N/A	N/A
**Intermediate (>5 to <=10 years)**	28,573 (37.8)	3,408 (24.2)	0.297	6,136 (40.3)	0.052	N/A	N/A	N/A	N/A	N/A	N/A	N/A	N/A
**Long-term (>10 years)**	32,699 (43.2)	2,203 (15.6)	0.635	2,924 (19.2)	0.537	N/A	N/A	N/A	N/A	N/A	N/A	N/A	N/A

[a]Standardized differences in comparison with immigrants
[b]Immigrants include economic immigrants and sponsored family immigrants,
[c]Resettled Refugees, include privately sponsored and government assisted refugees,
[d]Protected Persons includes successful asylum seekers and their dependents
[e]Immigrant -related characteristics of second generation immigrants/refugees are those of their mother/birthing parent
Small cells (<6) suppressed, and in the case of non-missing data other cells may be reported as ranges without percentage to reduce risk of re-identification in accordance with ICES policy
N/A= not applicable

© 2023 Brandenberger J et al. *JAMA Network Open.*

eTable 5. Crude and Adjusted Odds Ratios of Being Vaccinated (Full Cohorts) on April 24, 2022

	Children (N=1,096,756)		Adolescents (N=1,140,443)	
	**Crude odds ratio (95% CI)**	**Adjusted odds ratio (95% CI)**	**Crude odds ratio (95% CI)**	**Adjusted odds ratio (95% CI)**
**Younger age subgroup (Ref. older age subgroup)[a]**	0.71 (0.71-0.72)	0.64 (0.64-0.65)	0.74 (0.73-0.75)	0.75 (0.75-0.76)
**Male (Ref. female)**	0.99 (0.98-1.00)	0.98 (0.98-0.99)	0.91 (0.9-0.91)	0.90 (0.90-0.91)
**Immigration category (Ref. non-immigrants)**				
**Immigrants[b]**	1.28 (1.25-1.30)	1.30 (1.27-1.33)	0.97 (0.95-0.99)	1.10 (1.08-1.12)
**Resettled refugees[c]**	0.32 (0.31-0.34)	0.34 (0.33-0.36)	1.04 (1.00-1.09)	0.88 (0.84-0.91)
**Protected persons and others[d]**	0.48 (0.46-0.51)	0.55 (0.52-0.58)	0.92 (0.88-0.96)	0.99 (0.95-1.03)
**Second generation immigrant[b]**	0.92 (0.91-0.93)	0.98 (0.97-0.99)	1.10 (1.09-1.12)	1.12 (1.10-1.13)
**Second generation resettled refugee[c]**	0.38 (0.36-0.39)	0.42 (0.40-0.43)	0.74 (0.71-0.77)	0.72 (0.69-0.75)
**Second generation protected persons and others[d]**	0.52 (0.51-0.54)	0.63 (0.61-0.64)	0.93 (0.89-0.96)	0.98 (0.95-1.02)
**Rural (Ref. urban)**	0.76 (0.75-0.76)	0.72 (0.71-0.73)	0.70 (0.69-0.71)	0.7 (0.69-0.71)
**Material Deprivation Quintile (Ref. 1 - least deprived)**				
**2**	0.79 (0.78-0.80)	0.83 (0.82-0.84)	0.83 (0.82-0.85)	0.84 (0.83-0.85)
**3**	0.62 (0.62-0.63)	0.69 (0.68-0.70)	0.68 (0.67-0.69)	0.69 (0.68-0.70)
**4**	0.51 (0.50-0.52)	0.59 (0.59-0.60)	0.57 (0.56-0.57)	0.58 (0.57-0.59)
**5 (most deprived)**	0.38 (0.37-0.38)	0.47 (0.46-0.47)	0.42 (0.42-0.43)	0.44 (0.43-0.45)
**Neighbourhood COVID-19 Risk Decile (Ref. 10 – least at risk)**				
**1 (most at risk)**	0.68 (0.67-0.69)	0.64 (0.63-0.65)	0.89 (0.87-0.91)	0.79 (0.77-0.80)
**2**	0.9 (0.89-0.92)	0.78 (0.77-0.80)	1.02 (1.00-1.04)	0.87 (0.85-0.89)
**3**	0.89 (0.87-0.90)	0.71 (0.70-0.72)	0.99 (0.97-1.01)	0.79 (0.77-0.81)
**4**	0.91 (0.89-0.92)	0.76 (0.75-0.78)	0.94 (0.92-0.96)	0.79 (0.77-0.81)
**5**	1.10 (1.08-1.12)	0.85 (0.84-0.87)	1.16 (1.13-1.18)	0.91 (0.89-0.93)
**6**	1.12 (1.11-1.14)	0.88 (0.86-0.89)	1.08 (1.06-1.10)	0.86 (0.84-0.88)
**7**	1.27 (1.25-1.29)	0.92 (0.90-0.93)	1.17 (1.14-1.19)	0.87 (0.85-0.89)
**8**	1.28 (1.25-1.30)	0.95 (0.94-0.97)	1.26 (1.24-1.29)	0.98 (0.96-1.00)
**9**	1.13 (1.11-1.15)	1.03 (1.01-1.05)	1.14 (1.12-1.16)	1.08 (1.05-1.11)
**No influenza vaccination in 2019/2020 (Ref. influenza vaccinated)**	0.30 (1.29-0.30)	0.32 (0.31-0.32)	0.23 (0.22-0.23)	0.32 (0.31-0.33)
**Has a pediatric chronic condition (Ref. no chronic condition)**	1.17 (1.16-1.18)	1.13 (1.11-1.14)	1.44 (1.42-1.46)	1.30 (1.28-1.32)
**Primary care access model (Ref. rostered)**				
**Community Health Centre**	0.68 (0.66-0.69)	0.94 (0.92-0.97)	0.78 (0.76-0.81)	0.89 (0.86-0.91)
**Pediatrician**	1.37 (1.35-1.39)	1.17 (1.15-1.19)	1.46 (1.42-1.51)	1.27 (1.23-1.30)
**Non-comprehensive care**	0.78 (0.78-0.79)	0.85 (0.84-0.86)	0.93 (0.91-0.94)	0.92 (0.91-0.93)
**No regular care provider**	0.41 (0.40-0.41)	0.46 (0.45-0.46)	0.21 (0.21-0.21)	0.25 (0.24-0.25)
**No previous COVID-19 infection (Ref. previous infection)**	1.20 (1.17-1.23)	1.12 (1.09-1.14)	0.55 (0.53-0.57)	0.64 (0.62-0.66)

[a]sub-age groups for children are 4-7 and 8-10 and for adolescents are 11-14 and 15-17
[b]Immigrants include economic immigrants and sponsored family immigrants.
[c]Resettled Refugees include privately sponsored and government sponsored refugees
[d]Protected Persons and others includes successful asylum seekers and their dependents

© 2023 Brandenberger J et al. *JAMA Network Open.*

eTable 6. Adjusted Odds Ratios of Being Vaccinated Among First- and Second-Generation Immigrant and Refugee Children on April 24, 2022

	Immigrant/refugee children (N=53,000)	Second generation immigrant/refugee children (N=256,518)
**Variable**	**Odds ratio**	**Odds ratio**
**Age 4-7 (Ref. 8-10) years**	0.74 (0.71-0.77)	0.70 (0.68-0.71)
**Male (Ref. female)**	1.01 (0.97-1.05)	0.98 (0.96-1.00)
**Immigration category (Ref. immigrants[a])**		
**Resettled refugees[b]**	0.27 (0.25-0.29)	0.52 (0.49-0.54)
**Protected persons and others[c]**	0.41 (0.38-0.44)	0.63 (0.61-0.66)
**Recency of immigration (Ref. long term >10 years ago)**		
**Recent (0 to <=5 years)**	0.93 (0.74-1.18)	N/A
**Intermediate (>5 to <=10 years)**	0.92 (0.73-1.17)	N/A
**Region of origin (Ref. population average)**		
**Australasia & Oceania & Asia unspecified**	1.36 (1.08-1.72)	1.79 (1.53-2.10)
**Caribbean**	0.66 (0.58-0.76)	0.58 (0.56-0.61)
**Central Africa**	0.24 (0.16-0.35)	0.30 (0.25-0.35)
**Central America**	1.01 (0.85-1.20)	1.30 (1.23-1.38)
**East Africa**	0.88 (0.77-1.00)	0.59 (0.56-0.62)
**East Asia**	1.33 (1.23-1.45)	1.70 (1.65-1.75)
**Eastern Europe**	0.40 (0.35-0.46)	0.50 (0.48-0.52)
**Europe other**	1.12 (1.03-1.22)	0.95 (0.91-0.99)
**Middle East**	0.89 (0.83-0.95)	0.79 (0.76-0.81)
**North Africa**	0.54 (0.47-0.61)	0.58 (0.54-0.62)
**North America**	1.63 (1.51-1.76)	1.64 (1.55-1.74)
**South America**	2.37 (2.10-2.68)	1.49 (1.43-1.55)
**South Asia**	2.11 (2.00-2.22)	1.19 (1.16-1.22)
**Southeast Asia**	2.68 (2.47-2.92)	2.44 (2.37-2.52)
**Southern Africa**	1.23 (1.02-1.49)	2.23 (1.98-2.52)
**Western Africa**	0.69 (0.61-0.78)	0.72 (0.68-0.77)
**Rural (Ref. urban)**	0.49 (0.40-0.60)	0.54 (0.50-0.58)
**Material Deprivation Quintile (Ref. 1 - least deprived)**		
**2**	0.92 (0.86-0.98)	0.90 (0.88-0.93)
**3**	0.82 (0.77-0.87)	0.75 (0.73-0.78)
**4**	0.71 (0.66-0.76)	0.66 (0.65-0.68)
**5 (most deprived)**	0.65 (0.61-0.69)	0.56 (0.55-0.58)
**Neighbourhood COVID-19 Risk Decile (Ref. 10 – least at risk)**		
**1 (most at risk)**	0.37 (0.32-0.44)	0.49 (0.46-0.53)
**2**	0.49 (0.42-0.58)	0.64 (0.60-0.69)
**3**	0.43 (0.37-0.51)	0.60 (0.56-0.65)
**4**	0.47 (0.40-0.56)	0.64 (0.59-0.69)
**5**	0.52 (0.44-0.61)	0.67 (0.63-0.73)
**6**	0.54 (0.46-0.64)	0.71 (0.66-0.77)
**7**	0.62 (0.52-0.73)	0.78 (0.72-0.84)

© 2023 Brandenberger J et al. *JAMA Network Open.*

	Immigrant/refugee children (N=53,000)	Second generation immigrant/refugee children (N=256,518)
**Variable**	**Odds ratio**	**Odds ratio**
**8**	0.63 (0.53-0.75)	0.81 (0.75-0.88)
**9**	0.61 (0.50-0.74)	0.82 (0.76-0.90)
**Has a pediatric chronic condition (Ref. no chronic condition)**	1.23 (1.14-1.31)	1.16 (1.13-1.18)
**Primary care access model (Ref. rostered)**		
**Community Health Centre**	0.99 (0.88-1.11)	0.98 (0.91-1.06)
**Pediatrician**	1.10 (0.99-1.22)	1.34 (1.30-1.38)
**Non-comprehensive care**	0.85 (0.81-0.89)	0.94 (0.92-0.96)
**No regular care provider**	0.35 (0.32-0.38)	0.53 (0.51-0.54)
**No previous COVID-19 infection (Ref. previous infection)**	1.04 (0.94-1.15)	1.04 (0.99-1.09)

[a]Immigrants include economic immigrants and sponsored family immigrants.
[b]Resettled Refugees include privately sponsored and government sponsored refugees
[c]Protected Persons and others includes successful asylum seekers and their dependents
N/A = not applicable/available

eTable 7. Adjusted Odds Ratios of Being Vaccinated Among First- and Second-Generation Immigrant and Refugee Adolescents on April 24, 2022

	First generation (N=104,714)	Second generation (N=221,636)
**Variable**	**Odds ratio**	**Odds ratio**
**Age 11-14 (Ref 15-17)**	0.71 (0.69-0.73)	0.71 (0.69-0.72)
**Male (Ref. female)**	0.92 (0.89-0.95)	0.89 (0.87-0.91)
**Immigration category (Ref. immigrants[a])**		
**Resettled refugees[b]**	0.82 (0.77-0.86)	0.78 (0.74-0.82)
**Protected persons and others[c]**	0.98 (0.93-1.03)	0.94 (0.90-0.98)
**Recency of immigration (Ref. long term >10 years ago)**		
**Recent (0 to <=5 years)**	1.40 (1.34-1.47)	N/A
**Intermediate (>5 to <=10 years)**	1.2 (1.15-1.25)	N/A
**Region of origin (Ref. population average)**		
**Australasia & Oceania & Asia unspecified**	0.74 (0.60-0.92)	1.01 (0.84-1.22)
**Caribbean**	0.61 (0.57-0.65)	0.50 (0.48-0.52)
**Central Africa**	0.51 (0.45-0.59)	0.62 (0.55-0.71)
**Central America**	1.18 (1.03-1.34)	0.88 (0.83-0.94)
**East Africa**	0.93 (0.86-1.01)	0.93 (0.88-0.97)
**East Asia**	1.48 (1.38-1.58)	2.09 (2.01-2.19)
**Eastern Europe**	0.41 (0.38-0.43)	0.44 (0.42-0.46)
**Europe other**	0.81 (0.76-0.86)	0.74 (0.71-0.77)
**Middle East**	0.93 (0.89-0.97)	0.88 (0.84-0.92)
**North Africa**	0.94 (0.86-1.02)	1.00 (0.93-1.08)
**North America**	0.74 (0.70-0.79)	0.66 (0.61-0.71)
**South America**	1.85 (1.65-2.08)	1.31 (1.24-1.38)
**South Asia**	1.82 (1.74-1.90)	1.89 (1.83-1.95)
**Southeast Asia**	4.42 (4.10-4.77)	3.35 (3.18-3.52)
**Southern Africa**	0.79 (0.65-0.95)	1.26 (1.06-1.50)
**Western Africa**	1.07 (0.98-1.17)	0.95 (0.89-1.02)
**Rural (Ref. urban)**	0.57 (0.49-0.66)	0.56 (0.51-0.61)
**Material Deprivation Quintile (Ref. 1 - least deprived)**		
**2**	0.90 (0.85-0.95)	0.89 (0.86-0.92)
**3**	0.78 (0.74-0.83)	0.76 (0.73-0.78)
**4**	0.71 (0.67-0.75)	0.66 (0.64-0.69)
**5 (most deprived)**	0.61 (0.58-0.65)	0.54 (0.52-0.56)
**Neighbourhood COVID-19 Risk Decile (Ref. 10 – least at risk)**		
**1 (most at risk)**	0.58 (0.50-0.68)	0.81 (0.73-0.90)
**2**	0.57 (0.49-0.66)	0.87 (0.78-0.96)
**3**	0.59 (0.51-0.68)	0.75 (0.68-0.83)
**4**	0.54 (0.47-0.63)	0.79 (0.72-0.88)
**5**	0.64 (0.55-0.74)	0.91 (0.82-1.00)

© 2023 Brandenberger J et al. *JAMA Network Open.*

	First generation (N=104,714)	Second generation (N=221,636)
**Variable**	**Odds ratio**	**Odds ratio**
**6**	0.62 (0.53-0.72)	0.82 (0.74-0.91)
**7**	0.59 (0.51-0.69)	0.80 (0.72-0.89)
**8**	0.70 (0.60-0.81)	0.99 (0.89-1.10)
**9**	0.74 (0.62-0.88)	0.83 (0.74-0.93)
**Has a pediatric chronic condition (Ref. no chronic condition)**	1.34 (1.27-1.42)	1.36 (1.32-1.39)
**Primary care access model (Ref. rostered)**		
**Community Health Centre**	0.79 (0.73-0.86)	1.04 (0.94-1.14)
**Pediatrician**	1.11 (0.98-1.27)	1.29 (1.22-1.36)
**Non-comprehensive care**	1.02 (0.97-1.07)	1.05 (1.02-1.09)
**No regular care provider**	0.15 (0.15-0.16)	0.22 (0.21-0.22)
**No previous COVID-19 infection (Ref. previous infection**	0.55 (0.50-0.60)	0.54 (0.51-0.58)

[a]Immigrants include economic immigrants and sponsored family immigrants.
[b]Resettled Refugees include privately sponsored and government sponsored refugees
[c]Protected Persons and others includes successful asylum seekers and their dependents
N/A = not applicable/available

© 2023 Brandenberger J et al. *JAMA Network Open.*

eFigure 1. Study Population Inclusion Flowchart

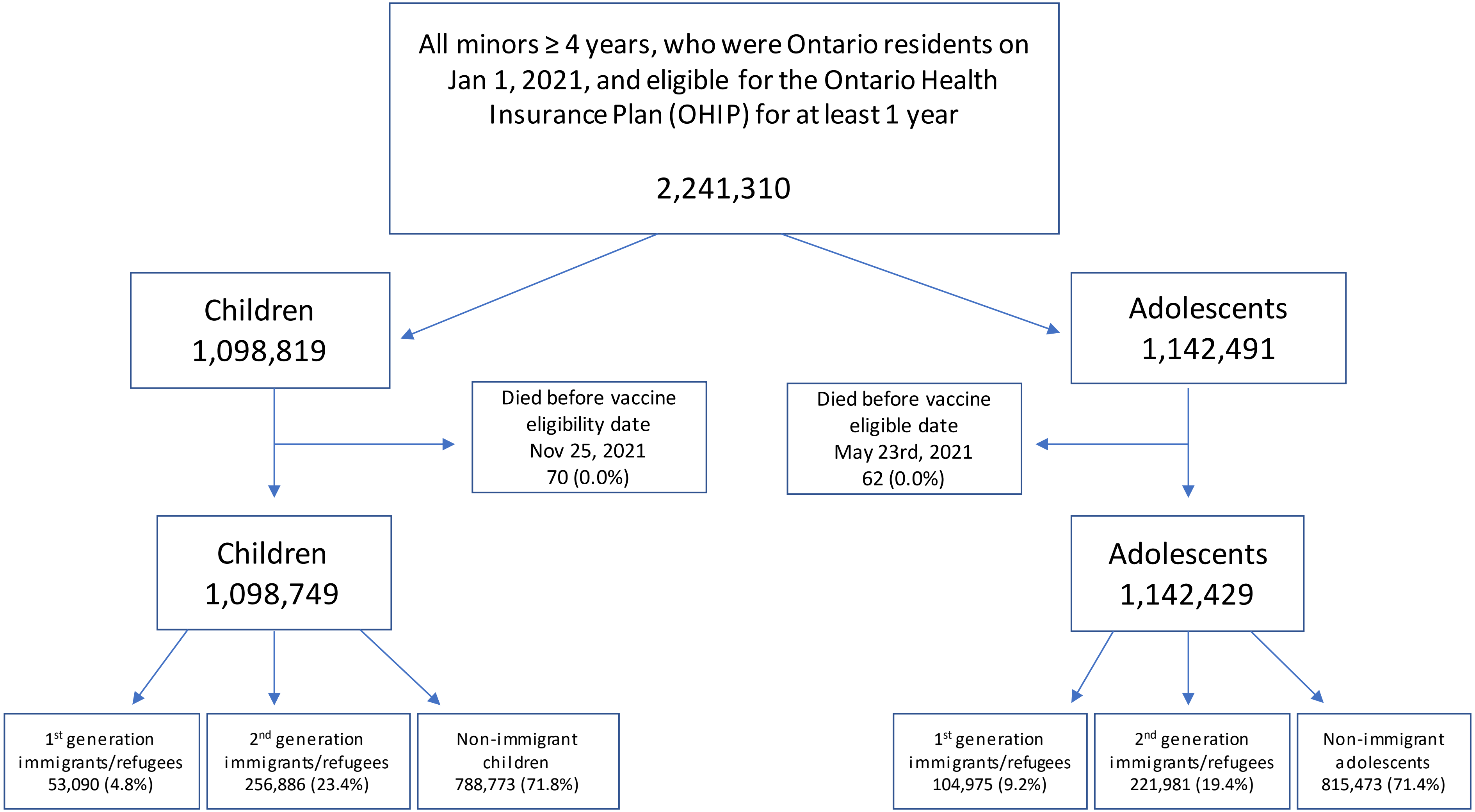

© 2023 Brandenberger J et al. *JAMA Network Open.*

eFigure 2. Milestones of the Ontario Vaccination Campaign in Relation to the Study Timeline

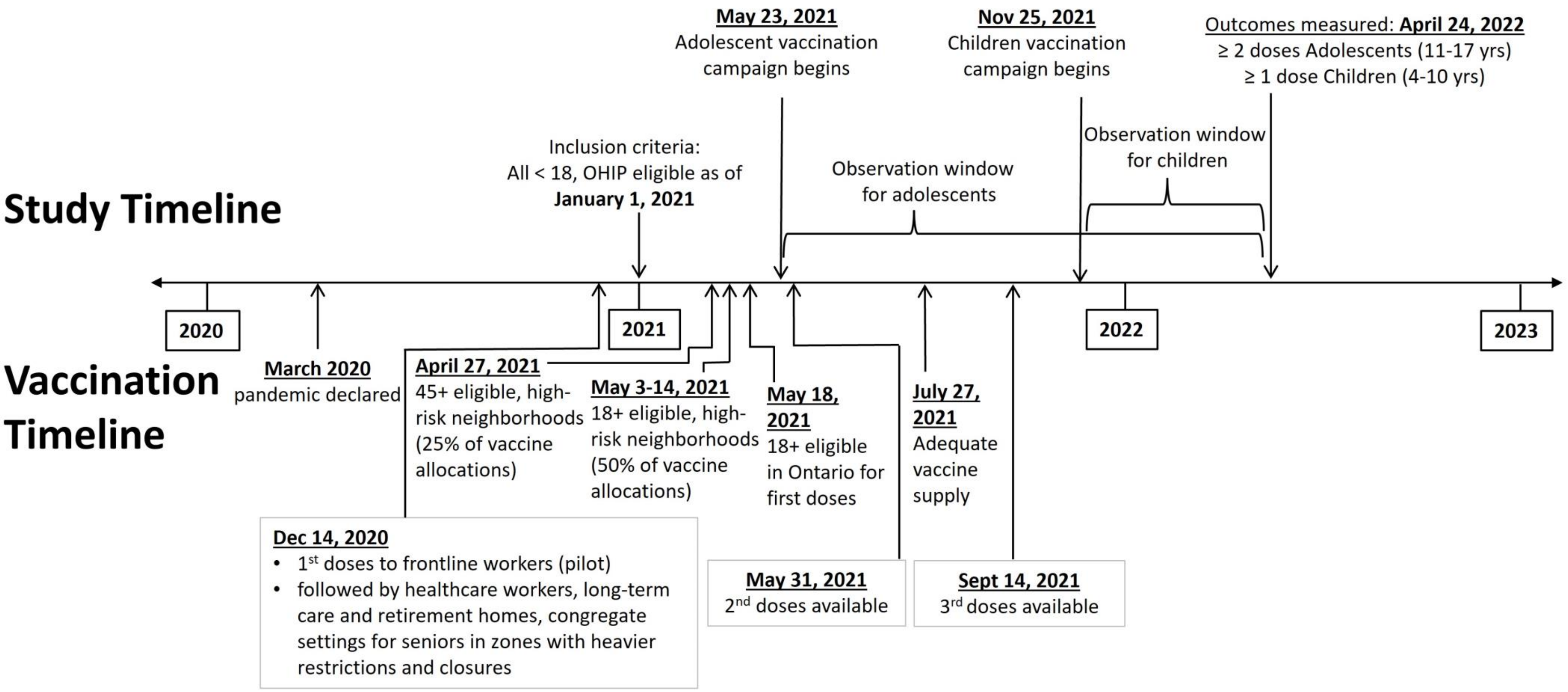

© 2023 Brandenberger J et al. *JAMA Network Open.*
